# Combined computational modeling and experimental study of the biomechanical mechanisms of platelet-driven contraction of fibrin clots

**DOI:** 10.1038/s42003-023-05240-z

**Published:** 2023-08-24

**Authors:** Christian Michael, Francesco Pancaldi, Samuel Britton, Oleg V. Kim, Alina D. Peshkova, Khoi Vo, Zhiliang Xu, Rustem I. Litvinov, John W. Weisel, Mark Alber

**Affiliations:** 1 Department of Mathematics, University of California Riverside, Riverside, CA 92521 USA; 2 Center for Quantitative Modeling in Biology, University of California Riverside, Riverside, CA 92521 USA; 3 Department of Microbiology and Immunology, University of Michigan, Ann Arbor, MI 48109 USA; 4 Department of Cell and Developmental Biology, University of Pennsylvania School of Medicine, Philadelphia, PA 19104 USA; 5 Department of Biomedical Engineering and Mechanics, Center for Soft Matter and Biological Physics, Virginia Tech, Blacksburg, VA 24061 USA; 6 Department of Pharmacology, University of Pennsylvania School of Medicine, Philadelphia, PA 19104 USA; 7 Department of Applied and Computational Mathematics and Statistics, University of Notre Dame, Notre Dame, IN 46556 USA; 8 Department of Bioengineering, University of California Riverside, Riverside, CA 92521 USA

**Keywords:** Computational models, Computer modelling, Computational biophysics, Translational research, Cell biology

## Abstract

While blood clot formation has been relatively well studied, little is known about the mechanisms underlying the subsequent structural and mechanical clot remodeling called *contraction* or *retraction*. Impairment of the clot contraction process is associated with both life-threatening bleeding and thrombotic conditions, such as ischemic stroke, venous thromboembolism, and others. Recently, blood clot contraction was observed to be hindered in patients with COVID-19. A three-dimensional multiscale computational model is developed and used to quantify biomechanical mechanisms of the kinetics of clot contraction driven by platelet-fibrin pulling interactions. These results provide important biological insights into contraction of platelet filopodia, the mechanically active thin protrusions of the plasma membrane, described previously as performing mostly a sensory function. The biomechanical mechanisms and modeling approach described can potentially apply to studying other systems in which cells are embedded in a filamentous network and exert forces on the extracellular matrix modulated by the substrate stiffness.

## Abstract (web summary)

A multiscale computational model quantifies the biomechanical kinetics of blood clot contraction driven by platelets interacting with and pulling on fibrin fibers and provides insights into the contractility of platelet filopodia.

## Introduction

**Fig. 1:**
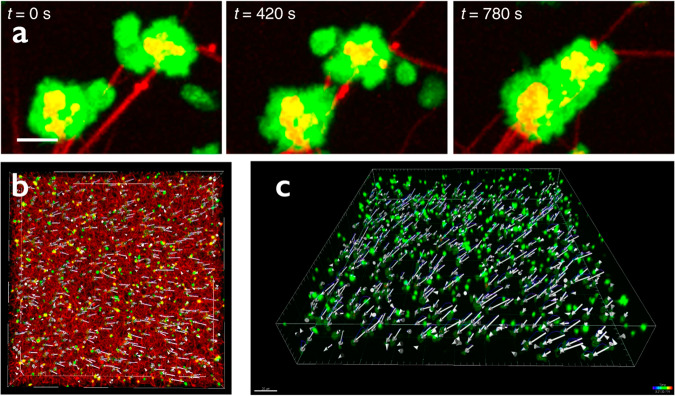
Clustering of the fibrin-attached neighboring platelets and deformation of the platelet-fibrin meshwork during contraction. **a** Serial confocal image showing the formation of secondary platelet clusters due to the approximation of platelet-bearing fibrin fibers during clot contraction. Platelets are green and fibrin is red. Scale bar: 5 μm. **b**, **c** Representative images of a three-dimensional platelet displacement field of a contracting clot; **b** top view, fibrin (red), platelet (green), and platelet displacement vectors are shown; **c** perspective view, platelets (green) with their displacement vectors are visualized (figure is reprinted from Kim et al. ^16^ under the conditions of the Creative Commons CC BY license).

Blood clots are gel-like structures that are formed at the sites of vessel injuries to stem the bleeding. Clots form as a result of combined cellular and enzymatic reactions that involve platelets and plasma components, including fibrinogen and other clotting factors. The major components of a blood clot include a 3D polymeric fibrin network with platelets attached to fibrin fibers and red blood cells embedded in the porous network. After formation, blood clots undergo volumetric shrinkage through a process called clot contraction or retraction, driven by activated platelets^1^ (see Fig. 1 which demonstrates local platelet clustering and a three-dimensional platelet displacement field of a contracting clot).

Intravital contraction of blood clots or thrombi has several important pathophysiological implications, such that it improves the sealing properties of hemostatic clots^2^, reduces clot size and improves blood flow past otherwise obstructive thrombi^3^, prevents clot rupture or thrombotic embolization^4^, and alters the susceptibility of blood clots to enzymatic lysis^5^. Accordingly, impairment of the clot contraction process is associated with life-threatening thrombotic conditions, such as ischemic stroke^3^, venous thromboembolism^4,6^, and others^7^. Recently, blood clot contraction was observed to be hindered in patients with COVID-19, especially in severe and fatal cases^8^.

Despite their clinical importance, the biomechanical mechanisms of clot contraction are not well understood. It is known that blood clot contraction is driven by traction forces generated by the platelet cytoskeleton that are transmitted to fibrin fibers via highly adhesive plasma membrane protrusions called filopodia^9^. One of the least studied aspects of clot contraction is the formation of platelet filopodia and their physical interaction with the fibrin network. The biomechanics of platelet-induced blood clot contraction is fundamental to understanding a multitude of other somewhat similar biological processes related to bidirectional cellular mechanotransduction, including cell motility, tissue regeneration and differentiation^10–14^, phagocytosis^9^, and cancer development^15^. Quantitative structural mechanobiology of platelet-driven blood clot contraction has emerged recently as a new avenue of biomechanics^16^ and can provide a foundation for understanding the dynamic and complex biomechanical interplay between non-muscle cells and the fibrous extracellular matrices of various compositions.

The basic cellular biomechanical mechanisms of the contraction process were not elucidated until our paper in 2017^16^. In particular, we are not aware of any paper(s) that have investigated the role of extension and retraction of platelet filopodia in clot contraction until we first described this mechanism^16^. Unlike in other cell-mediated physiological processes^9^, the mechanism driving clot contraction has not been well-known and commonly accepted—neither the most relevant papers on platelet biomechanics^17–22^ nor one of the latest and most comprehensive review articles on clot contraction^23^ discuss the role of platelet filopodia in clot contraction. Therefore, the role of extending and contracting platelet filopodia as the driving force of fibrin network densification is not entirely clear, and worth further investigation, including the modeling approach that enables us to glean mechanistic and quantitative information about filopodia dynamics not available experimentally.

In this paper, a combination of 3D computational model simulations and experimental observations is used to provide important mechanistic insights into different processes involved in the contraction of blood clots. Model simulation results quantify microscale mechanisms of platelet–fibrin fiber interactions involved in platelet clustering and macroscale clot contraction. While the model allows for platelet–platelet interaction, these appear only once the network has been sufficiently compacted and fibrin-attached platelets are moved passively in proximity to each other. When this happens, platelets start to adhere to each other and form secondary clusters. Active platelet–platelet pulling interactions followed by their compact packing may be essential during the contraction of fibrin-free hemostatic platelet plugs formed in some experimental models of intravital vessel wall injury^24–26^. Contraction of such fibrin-free platelet aggregates is beyond the scope of this work, although elements of our model are potentially applicable to the mechanical and structural rearrangement of aggregated platelets in the absence of fibrin.

Many characteristic features of blood clots and their dynamic properties cannot be measured experimentally at this time. For example, platelet activation can involve the formation of filopodia in each platelet, the attachment of individual filopodia to fibrin fibers, generation of pulling forces and the retraction of filopodia, shortening of fibrin fibers and network densification, etc. Segregation and quantification of each of these elementary steps of the clot contraction process are hardly feasible, but these features can be studied independently, as well as in concert, using a multiscale computational approach. Computational modeling offers the possibility to analyze quantitatively each structural and mechanical component of a blood clot and integrate the resulting impact on the contraction of a whole clot.

This work aims at producing an understanding of how activated platelets drive clot contraction and influence the overall dynamics of the process through alterations in the traction forces exerted by their filopodia, which are the main structural and mechanically active elements of a contracting platelet. A major concept underlying the model presented in this paper is the existence of biomechanical feedback between the contractility of individual platelets (or filopodia) and the strain-stiffening of the fibrin matrix (or individual fibrin fibers)^9,14,15^. In particular, the model developed by our group employs a 3D computational modeling approach, in which the extent of platelet activation can be represented by altering the number of filopodia per platelet and the magnitude of the force exerted by each filopod. Importantly, the filopodia-generated traction force is modulated by the mechanical response to changing the stiffness of fibrin fibers under tension. This methodology allows for the quantitative representation of different platelet activation mechanisms at the microscopic scale and prediction of the resulting bulk properties of the blood clot, such as the extent and kinetics of clot contraction at the macroscopic level (see the section “Model description”).

Mechanical properties of blood clots have been extensively studied and shown to depend on the viscoelasticity of their major components, that is fibrin, platelets, and red blood cells, as well as clot structural and mechanical interplay^7,17,27–31^. Fibrin plays a key role in providing mechanical stability to a clot by forming a network, a scaffold that propagates external stresses generated by blood flow or extravascular forces, as well as by traction forces of activated platelets during clot contraction^29,32–34^. Fibrin networks reveal a remarkable stiffening behavior upon stretching and shear and a softening–stiffening transition under compression^29,32–34^. Individual fibrin fibers exhibit elastomeric behavior, indicating low elastic modulus (~MPa) while being extremely extensible (>300% strain)^35^. The propensity of fibrin clots to rupture has been studied using thermodynamic and structural analysis^36^ and quantified in terms of the critical energy release rate^37,38^.

Platelets play a key role in clot contraction or clot volume shrinkage by exerting traction forces on surrounding fibers causing compaction of fibrin in the vicinity of individual platelets, leading to local densification of fibrin in a clot that dramatically increases network density and clot stiffness^16^. The average maximum contractile force generated by an individual platelet was measured to be 29 nN^17^. Clot contraction is accompanied by dramatic structural rearrangements so that red blood cells are packed tightly in the interior part, while a meshwork of fibrin and platelets accumulates at the exterior of the clot^2,39^. These structural changes result from a physical interplay between mechanically active contractile platelets and passive viscoelastic elements (red blood cells, fibrin) of the complex and dynamic biomechanical system^16,40^. Clot contraction has several kinetic phases that depend on the molecular and cellular composition of the blood in which a clot is formed^41^. The presence of red blood cells (RBCs) alters clot contraction kinetics and results in the formation of tightly packed polyhedral-like erythrocytes (polyhedrocytes) forming an impermeable barrier in contracted clots and thrombi^42^.

Previous theoretical and computational models of blood clot contraction, while investigating the role of major components and important mechanical aspects^26,40,43–49^, did not provide a description of structural details, such as fibrin network organization and individual platelets embedded in the network^50^. Despite important thermodynamic insights^40^, the driving forces and mechanisms of structural rearrangement of the contracting blood clots are not well understood. Several computational models were used recently to study these processes^18,51^ especially focusing on the asynchronous or asymmetric behavior of platelets in a contracting clot. Also, previous work^43^ introduced an important coarse-grained molecular dynamic particle-based model for studying the formation of an individual filopod during the early stages of platelet activation.

The main novelty of the three-dimensional (3D) multiscale computational modeling approach described in this paper is in its detailed representation of platelet functionality involving filopodia at various degrees of activation, with corresponding local microscale structural and mechanical alterations that have a cumulative macroscale impact on the contraction of the whole blood clot. The model combines a microscale sub-model of the “pulling hand-over-hand on a rope” mechanism of a filopod pulling on a fibrin fiber based on the fiber’s stiffness, with sub-models of an individual fibrin fiber and whole fibrin network. Also, in this paper, we study the contraction process with varying numbers of filopodia per platelet, since filopodia have been previously thought of as mostly performing a sensory function^52,53^, while lamellipodia are more commonly involved in force generation.

Specifically, the current work describes in detail the remodeling of the fibrin network and the accumulation of fibrin near the surface of each activated platelet during clot contraction. Also, most previous models of mechanics of biogels were designed to simulate the stretching and deformation of biogels by forces applied externally on their surfaces. (For an example of a model describing the stretching of a fibrin network by external forces see ref. ^30^.) To the best of our knowledge, the model, described in the current paper, is the first one to implement in detail internal forces exerted individually by a large number of cells (activated platelets) positioned inside of a network and pulling at individual fibers and locally condensing fibrin and contracting the network. This introduces complexity and biological relevance, makes it possible to calibrate the model using specific experimental data, and results in specific predictive model simulations.

## Results

### Model description

**Fig. 2:**
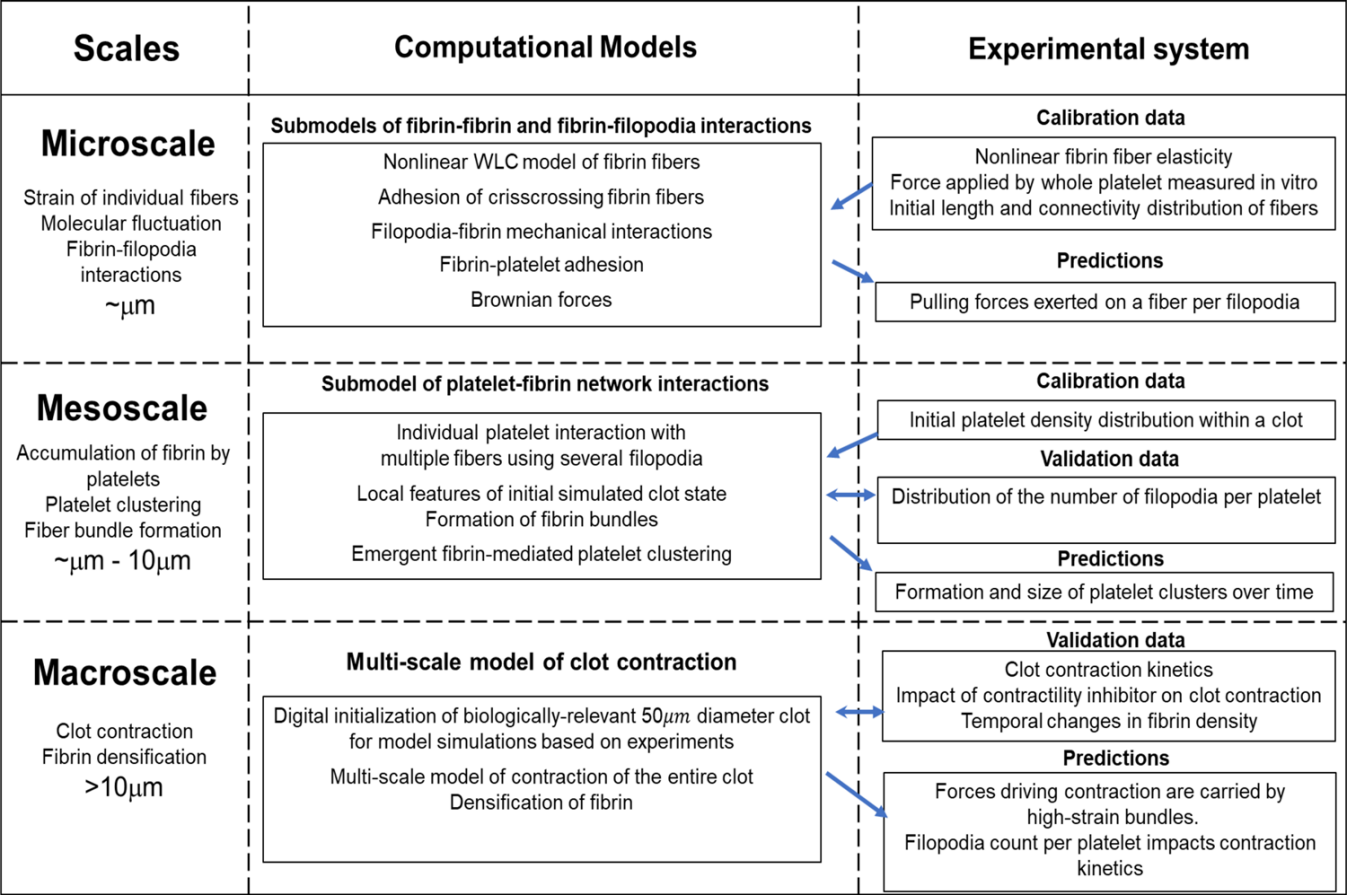
Diagram of a 3D multiscale modeling framework and exchange of calibration and validation data leading to predictive simulations. Microscale and mesoscale submodels of smaller-scale phenomena are assembled together as components and then calibrated to create a model capable of simulating data at the micro, meso, and macro scales.

**Fig. 3:**
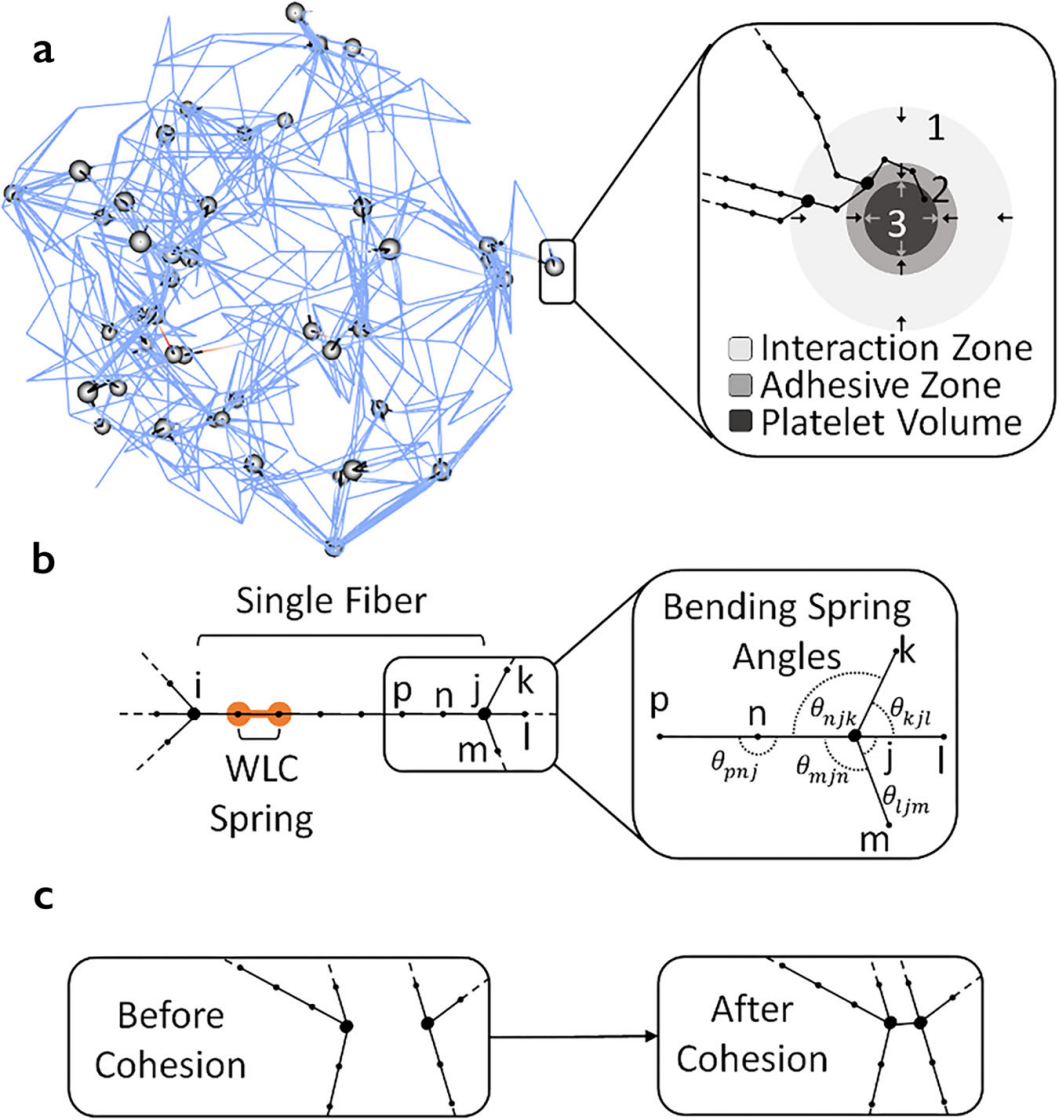
Diagram of the components of the 3D model of clot contraction. **a** Snapshot of a model representation of a fibrin network (*lines*) and platelets (*spheres*). (inset) Zones of influence include filopodia interaction zone (region 1), Adhesive Zone (region 2), and a platelet volume zone representing the platelet body (region 3). Arrows indicate if the forces applied to the fibers act toward the center of the platelet or away from the center. **b** A single fibrin fiber is modeled using several Worm-Like-Chain (WLC) sub-segment springs and bending springs. Segmentation of fiber into several springs is indicated by sub-nodes. Here points *i* and *j* are the main nodes (e.g. branching points, bigger black nodes) for the fiber, while the points between *i* and *j* (e.g. *p* and *n*) are computational sub-nodes, and the segments between sub-nodes are the WLC spring sub-segments (see example with orange halo). **c** Diagram of the formation of a cohesive bond between sufficiently close fibers (**b** and **c** were modified from literature^30^ with permission from Elsevier to include images of new model components).

The diagram in Fig. 2 provides a description of the coupling of all sub-models at specific scales into a three-dimensional (3D) multiscale computational framework for simulating clot contraction. Figure 2 also provides specific predictive results obtained by using a combination of biologically calibrated model simulations and experiments. Specific ranges of parameter values are provided in Supplementary Table 1. This multiscale model combines a 3D model of a fibrin network with a detailed sub-model representing a filopod of an individual platelet pulling on a fibrin fiber and locally condensing it (Fig. 3a inset). Individual fibrin fibers are described using a mass and spring modeling approach, which includes fiber stretching, bending (Fig. 3b), and fiber–fiber cohesive interactions (Fig. 3c) (see also Supplementary Note 1 for additional model details and Table 1 for ranges of parameter values). The initial configuration of model fibrin fibers and platelets are arranged to represent a 23.8 µm-radius spherical clot (see Supplementary Note 2). These configurations are generated to preserve experimentally measurable properties such as fibrin and platelet density, and a portion of platelet co-localized fibrin (see Supplementary Notes 2.1–2.3).

**Table 1:** Model simulation parameters.

Variable or parameter^a^	Value
*Fibrin simulation parameters*	
Monomers per fiber, ${N}_{\rm {{m}}}$	200–1100^54^
Monomer persistence length, ${L}_{\rm {{p}}}$	0.005–1.074 nm^54^
Fibrin radius, ${r}_{{\rm {{fib}}}}$	0.05 μm^30^
Temperature, $T$	300 K
Normalized contour length multiplier, $C$	2.35^54^
Fibrin volume fraction	0.3%^65^
Fibrin network radius	23.8 µm
Platelet simulation parameters	
Platelet radius, ${r}_{\rm {{p}}}$	1.1 μm^105^
Adhesive zone fraction, ${f}_{\rm {{r}}}$	0.9
Platelet range, ${r}_{{\rm {{fil}}}}$	2.2 μm^16^
Adhesion zone force, ${F}_{0}$	4 nN^58–60^
Average platelet density	8 × 10^5^ platelets/mm^3 ^^64^
Fibrin fraction initialized near platelets, ${P}_{{\rm {{fnp}}}}$	0.5 (see Supplementary Note 2.1)
Contraction time	30 min
Number of filopodia per platelet	Discretized truncated lognormal distribution (see the section “Model validation” and Fig. 4)^106^.
*Platelet contractile force-fitted parameters*^17^	
${f}_{0}$, force of platelet for zero stiffness	2.2 nN
$a$, fitting parameter	61
$b$, fitting parameter	3.6
$c$, fitting parameter	132
$d$, fitting parameter	1.4 nN
$\tau$, force relaxation time	637 s

^a^The mathematical symbol to represent the variable in equations is shown in italics.

#### Fibrin network sub-model

Based on the previous model^30^ developed by our group, each fibrin fiber is represented as a segment between two nodes (branching points in a 3D fibrin network) and is divided by sub-nodes (virtual arbitrarily created points) into equally spaced sub-segments of length 0.3 µm modeled as non-linear Worm-Like-Chain elastic springs (Fig. 3b). Consecutive sub-segments also have bending forces modeled through linear bending springs. Finally, fiber segments can form cohesive bonds (crisscrossing or oblique contacts) when two non-parallel fibers/segments get in close proximity and touch each other (Fig. 3c, see Supplementary Note 1.1 and previous work^30^ for more details).

The dynamics of fiber nodes and sub-nodes are described by a Langevin equation in an overdamped regime:

1 $${\eta }_{{\rm {f}}}{\dot{x}}_{{\rm {f}},j}={F}_{{\rm {f}},j}+{F}_{{\rm {f}},j}^{{\rm {B}}}$$

where ${x}_{{\rm {f}},j}$ is the position vector of the *j*th fiber node, ${F}_{{\rm {f}},j}$ is the deterministic force acting on the *j*th fiber node or sub-node (fibrin elastic forces, bending forces, and fibrin–platelet interaction forces, discussed below; see Supplementary Note 1.1 for details), ${\eta }_{{\rm {f}}}$ represents the drag coefficient for a fiber node in liquid serum, and ${F}_{j}^{{{B}}}$ is the Brownian force due to thermal fluctuations (see Supplementary Notes 1.1 for more details). The formation of cohesion bonds instead of the volume exclusion principle in our model prevents fibrin fibers from crossing each other (see Supplementary Note 1.1 and ref. ^30^ for more details).

The spring constant *B*_s_ of the bending springs is calculated using the relation *B*_s_ = *EI*, where *E* is Young’s modulus, which was determined by fitting the Worm-Like-Chain forces to AFM data from literature^54^ (see Supplementary Fig. 1). Parameter $I$ is the moment of inertia of a circle. See Supplementary Note 3.1 for more details of the calculation, which leads to *B*_s_ = 2.252 × 10^−3^ nN μm^2^.

The relatively small mass of the fibrin and platelets, and the relatively viscous surrounding media (i.e. low Reynolds’ number (<<1) environment) makes it reasonable to assume that the dynamics of individual nodes are overdamped as in ref. ^30^, and so the inertia term is omitted in the system (1). The system (1) is then discretized and solved using a forward Euler scheme:

2 $${x}_{{\rm {f}},j}^{n+1}={x}_{{\rm {f}},j}^{n}+\frac{{{\rm {d}}t}}{{\eta }_{{\rm {f}}}}\left({F}_{{\rm {f}},j}^{n}+{F}_{j}^{n,{\rm {B}}}\right).$$

A more detailed discussion of the model forces, coefficients, and calibration is provided in Supplementary Notes 1.1 and 1.3.

#### Platelet and filopodia sub-models

Each platelet interacts with fibrin fibers through adherent protrusions called filopodia, leading to clot contraction as filopodia pull fibrin fibers toward the cell body. This, in turn, leads to remodeling of the fibrin network, causing fibrin accumulation near the surface of platelets and densification of the fibrin network, which are the most important structural features of clot contraction. As a result of fibrin network compaction, some fibrin-attached platelets are moved passively close enough to each other and form secondary clusters.

The dynamics of platelets are described in terms of the following Langevin equations. Namely, the motion of the center of mass of the *i*th platelet is described by the following ordinary differential equations:

3 $${m}_{{\rm {p}},i}{\ddot{x}}_{{\rm {p}},i}={F}_{{\rm {p}},i}-{\eta }_{{\rm {p}}}{\dot{x}}_{{\rm {p}},i}+{F}_{{\rm {p}},i}^{{\rm {B}}}$$

where ${F}_{{\rm {p}},i}$ is the sum of the deterministic forces applied to the *i*th platelet. These forces include fibrin-platelet or platelet–platelet interactions (described below, and more details are in Supplementary Notes 1.1–1.3). The viscous damping force, due to the clot being filled with serum, is represented by ${\eta }_{{\rm {p}}}{\dot{x}}_{{\rm {p}},i}$ and ${F}_{i}^{{\rm {B}}}$ denotes the Brownian force due to thermal fluctuations^55^ (see also Supplementary Note 1.1 for details). The system is assumed to be in an overdamped regime as in Britton et al. ^30^. The inertial term is therefore neglected, and the system (3) is discretized in time using a Forward Euler scheme:

4 $${x}_{{\rm {p}},i}^{n+1}={x}_{{\rm {p}},i}^{n}+\frac{{{\rm {d}}t}}{{\eta }_{{\rm {p}}}}{\left({F}_{{\rm {p}},i}^{n}+{F}_{i}^{n,{{{\rm {B}}}}}\right)}$$

where the superscripts $n$ and $n+1$ refer to the vector quantitates at time steps $n$ and $n+1$ for the *i*th platelet and ${{\rm {d}}t}$ is the time step; ${\eta }_{{\rm {p}}}$ represents the drag coefficient for a platelet with fixed volume in plasma.

#### Filopodia contractile forces

**Fig. 4:**
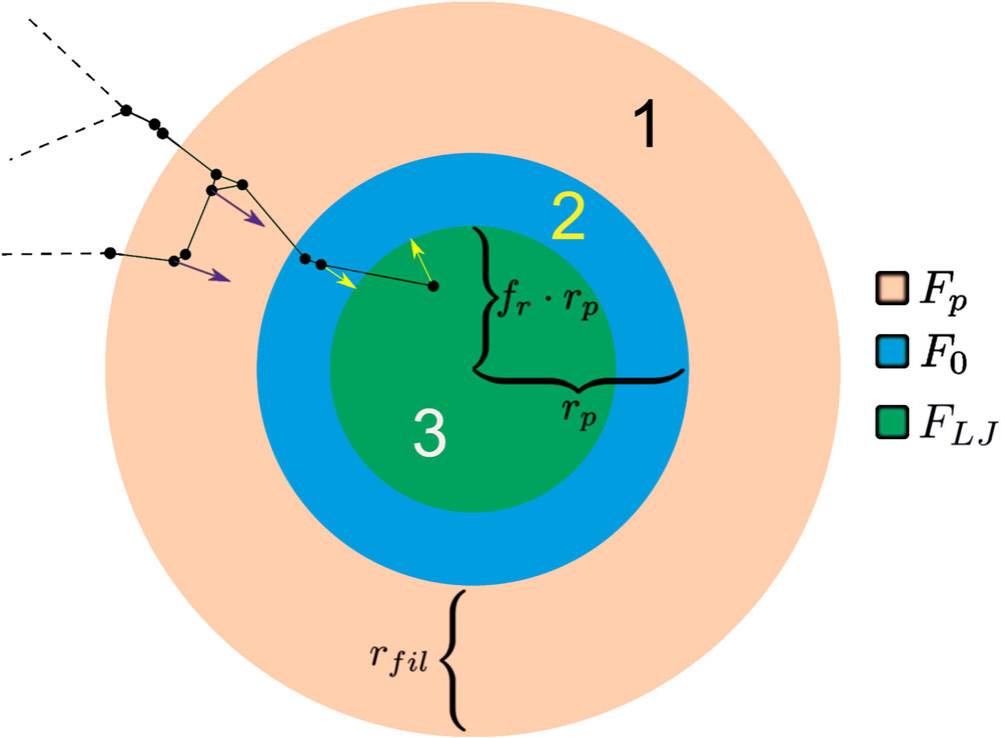
Platelet–fiber interaction schematic. Schematic of a model representation of an individual platelet interacting with fibrin fibers (black lines) in a clot. Different forces (*arrows*) are applied to fiber nodes or sub-nodes (*black dots*) depending on the distance from the platelet center of mass. The navy arrows represent the platelet’s filopodia pulling on randomly selected fiber nodes within ${r}_{{\rm {{fil}}}}$ of the platelet surface (i.e., within the filopodia interaction zone, region 1). Here ${r}_{\rm {{p}}}$ is the radius of the (spherical) platelet and the fraction ${f}_{\rm {{r}}}=0.9$ is used to distinguish the Adhesive Zone which pulls fibrin in (region 2) with adhesive forces (yellow arrow pulling with force ${F}_{0}$ from the platelet body (region 3) which volume-excludes fibrin by pushing out via a Lennard Jones potential ${F}_{{\rm {{LJ}}}}$. Detailed expressions for the forces are provided in Supplementary Note 1.1, Eqs. (5)–(8).

Filopodia of activated platelets are cytoskeleton-driven cell membrane protrusions that can attach to fibrin fibers and pull them toward the platelets^16^. The fibrin–platelet binding and traction are the most important interactions in the model simulations and the main mechanism driving clot contraction. In the model, the body of each activated platelet is represented as a sphere of radius ${r}_{\rm {{p}}}$ (Fig. 4, regions 2 and 3). Additionally, the platelet extends filopodia in the region between the surface of the platelet body (sphere of radius ${r}_{{\rm {p}}}$) and the surface of a sphere of radius ${r}_{{\rm {p}}}+{r}_{{{\rm {fil}}}}$ (Fig. 4, region 1, also known as the filopodia interaction zone), where ${r}_{{{\rm {fil}}}}$ represents the maximum length of filopodia in simulations. Filopodia can attach to fibrin fibers and other platelets within the filopodia interaction zone. A spherical geometry is used because platelets are non-polar cells, hence shape changes and forces exerted by activated platelets on their nearby environment are isotropic.

**Fig. 5:**
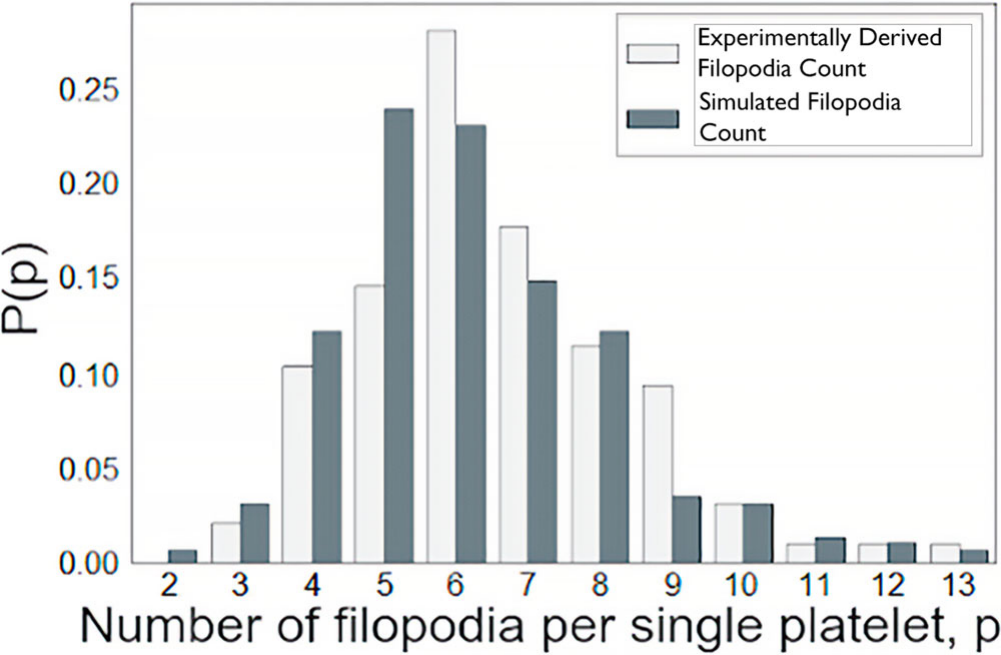
Probability distributions of active filopodia per platelet. The probability density functions of numbers of filopodia per platelet in 10 simulated clots were validated using specific experimental data and in four experimentally obtained platelet–fibrin meshworks.

Each platelet in the model has several filopodia varying between 2 and 13 (maximum number observed in experiments^16^, see also Fig. 5). The number for every individual platelet is determined by a discrete random variable generated from a truncated log-normal distribution obtained through fitting with experimental counts of filopodia per activated platelet (see Fig. 5). Each filopod can only connect with one fibrin fiber, thus pulling on a node or sub-node during contraction. This happens if a fibrin node or sub-node enters the near-platelet region where filopodia can extend (Fig. 4, region 1) and at least one filopod is available but not yet attached. In the simulations, a node or sub-node on a fibrin fiber is considered to enter this region if the distance between the center of mass of the node or sub-node and the platelet ${d}_{{\rm {{fp}}}}$ satisfies ${{r}_{\rm {{p}}} \, < \, {d}_{{\rm {{fp}}}} \, < \, r}_{\rm {{p}}}+{r}_{{{\rm {fil}}}}+{r}_{{{\rm {fib}}}}$, where ${r}_{{{\rm {fib}}}}=0.05\,{\rm {\mu {m}}}$ is the radius of a fibrin fiber^30,54,56^. In the model hundreds of fibers are simulated corresponding to hundreds of segments, summing up to tens of thousands of fibrin nodes and sub-nodes, while the platelet density used corresponds to 45 platelets in the simulations, making fibrin-platelet interactions much more likely than platelet–platelet interactions for the clot contraction simulated with the model.

When a filopod attaches to a fibrin node or sub-node forces are applied along the direction connecting the centers of mass of the platelet extending the filopod and the fibrin node or sub-node. The force exerted by the filopod is applied to the center of mass of the fibrin node or sub-node toward the center of mass of the platelet extending the filopod (see arrows in Fig. 4). A corresponding reaction force is also applied to the center of mass of the platelet extending the filopod in the opposite direction. Fiber nodes or sub-nodes are pulled by filopodia towards the platelet with a force, ${F}_{\rm {{p}}}$, when they are in the filopodia interaction zone (Fig. 4, region 1). A constant adhesion force (directed toward the platelet), ${F}_{0}$, representing the adhesion between fibers and platelet surface, is applied to fiber nodes or sub-nodes when they enter the Adhesive Zone (region 2). A Lennard–Jones repelling force, ${F}_{{\rm {{LJ}}}}$ is applied to the fiber nodes and sub-nodes, and platelets when they enter the Platelet Volume (region 3) to enforce volume exclusion preventing platelets or fibrin fibers from overlapping with the given platelet. When fibers enter this region the Lennard–Jones force pushes them away from the center of mass of the platelet.

Model simulations are performed under the assumption that once a filopod attaches to a fibrin fiber, the pulling force of a filopod, ${F}_{{\rm {p}}}$, depends on the local stiffness of the fiber at each pulled node or sub-node. Specifically, fibrin is a strain-stiffening material. Therefore, in our model, the average local strain of the fiber at the node or sub-node being pulled is used to calculate the local stiffness (see Supplementary Note 3.1 and Supplementary Table 2 for single fiber sub-model calibration).

For each node or sub-node of a fiber pulled by a filopod, the local fiber strain, $\bar{\gamma }$, is calculated as the average strain of all sub-segments that include that node or sub-node. Then $\bar{\gamma }$ is the fiber strain used by the filopod to produce a pulling force. The average local stiffness of a fiber attached to a filopod (node or sub-node), is calculated as follows: $k=\frac{{\rm {d}}{F}_{{{\rm {WLC}}}}(\bar{\gamma })}{\bar{d\gamma }}$.

The following formula has been fitted to experimental data to approximate the contractile force exerted by an entire platelet:

5 $${F}_{{{\rm {platelet}}}}\,(t,k)={f}_{0}+(1-{\rm {d}}{{\rm {e}}}^{-t/\tau })\frac{{ak}}{{bk}+c}.$$

Here, $t$ is the time evolved from the beginning of contraction (i.e., the start of simulation) of the clot and ${f}_{0},{a},{b},{c},{d},\,\tau$ are parameter values determined by fitting experimental data^17^ (see Supplementary Note 3.2, Supplementary Table 3, Supplementary Fig. 3, and Table 1 for further details and parameter values used in simulations). Formula (5) considers both previously observed exponential loading time^17^ and the monotonic increase and saturation of the contractile cellular force. This is similar to what was observed in ref. ^57^, where stem cell forces were shown to have a comparable dependence on the stiffness of their surrounding extracellular matrix. However, the present model includes a sub-model representing the pulling force of a single filopod, and no experimental data characterizing such force is available at this time. Instead, we tested several hypothesized expressions to derive and calibrate the pulling force of a single filopod ${F}_{{{\rm {filopod}}}}$ from ${F}_{{{\rm {platelet}}}}$ (see for details Supplementary Note 3.2; Comparison with experimental data is provided in Supplementary Note 4.3).

The function of the filopod pulling force that produced the best and very good agreement with experimental data is given by the following formula:

6 $${F}_{\rm {filopod}}\,(k)=\left({f}_{0}+\frac{{ak}}{{bk}+c}\right)/2.$$

During filopodia contraction, the applied force acts on fibrin nodes or sub-nodes on a fibrin fiber only while ${{r}_{{\rm {p}}} \, < \, {d}_{{{\rm {fp}}}} \, < \, r}_{{\rm {p}}}+{r}_{{{\rm {fil}}}}+{r}_{{{\rm {fib}}}}$. When these conditions are not satisfied, the filopod detaches from the fibrin fiber. For platelet–fibrin interactions, if the distance between fibrin nodes, sub-nodes, and a platelet instead satisfies ${{d}_{{{\rm {fp}}}} \, < \, r}_{{\rm {p}}}$, first an adhesive force and then a volume exclusion force are applied (detailed in the next subsection). Additionally, following the interaction between a filopod and a fibrin fiber, once the filopod is detached it re-attaches and starts pulling on the next node or sub-node on the fibrin fiber. This modeling component reproduces previously observed the “pulling hand-over-hand on a rope” mechanism^16^. If no such new fibrin node or sub-node exists (e.g., terminal node of a fiber), the filopodia are left free and fluctuate to connect with other available fibers.

There is currently no experimental evidence to distinguish between subsequent pulling on a fiber by different platelet filopodia and repeated pulling on a fiber by the same filopod. However, it is unlikely that this difference in filopodia pulling is critically important for clot contraction from a biomechanical viewpoint since platelets in both cases perform the same amount of work to shorten and compact fibers, which therefore does not affect the results of the model.

#### Platelet surface adhesion and platelet volume mechanics

During clot contraction, each platelet pulls fibrin fibers toward its center of mass. In the model, a fraction of the platelet radius, ${f}_{\rm {{r}}}=0.9$, is used to denote the boundary of the Adhesive Zone. If the distance between a platelet center and a fibrin node or sub-node satisfies ${{f}_{{\rm {r}}}\cdot r}_{{\rm {p}}}+{r}_{{\rm {f}}} \, < \, {d}_{{{\rm {fp}}}} \, < \, {r}_{{\rm {p}}}+{r}_{{\rm {f}}}$, then the fiber is within the Adhesive Zone surrounding the platelet. When a fibrin fiber is within the Adhesive Zone of an activated platelet, the model applies a fixed constant force, ${F}_{0}$, to the fibrin nodes’ or sub-nodes’ centers of mass directed toward the center of mass of the platelet. The adhesion zone force is a part of the platelet pulling generated by the platelet actomyosin machinery, by which contractility is transmitted to fibrin fibers via the platelet integrins. The contractile force has been measured for individual platelets using different experimental methods described in refs. ^58–60^. We estimated the minimal value of ${F}_{0}$ from Supplementary Fig. 2 to be 4 nN. The adhesive zone for the platelets is shown in Figs. 3a and 4 (region 2). This force tends to move fiber fibers in the Adhesive Zone closer to the center of mass of the platelet. Once ${{d \, < \, f}_{{\rm {r}}}\cdot r}_{{\rm {p}}}+{r}_{{\rm {f}}}$, for fiber nodes and sub-nodes 3D mechanics needs to be considered. Specifically, to reflect the role of a platelet body, a repulsive force is introduced that pushes fibrin fibers away from the platelet. This type of force is often referred to as a volume exclusion force^61^ and is applied to the fibrin nodes and sub-nodes in the opposite direction, i.e. away from the interacting platelet. This Platelet Volume zone is illustrated by region 3 in Figs. 3a and 4. The force magnitude is calculated using a standard Lennard–Jones potential-based force ${F}_{{{\rm {LJ}}}}=12\epsilon ({\sigma }^{12}/{x}^{13}-{\sigma }^{6}/{x}^{7})$, where $\sigma =2{{f}_{{\rm {r}}}r}_{{\rm {p}}}/{2}^{1/6}$, $\epsilon =16\,{\rm {{nN}}}\cdot {\rm {\mu m}}$ and $x={d}_{{{\rm {fp}}}}\,{{\rm {or}}}\,{d}_{{{\rm {pp}}}}$ (see also Supplementary Note 1.1 for details on the parameter). Since the model framework may potentially leave some filopodia unengaged, and the number of filopodia per platelet was calibrated to the number of actively pulling filopodia, it is important to note that in our simulations all available filopodia were actively engaged with pulling fibrin fibers.

#### Computational model implementation

Several computational techniques were introduced to speed up and optimize a very large number of model simulations. First, the simulation code was implemented using CUDA for fully utilizing GPU parallelization. All independent operations are performed in parallel using the CUDA Thrust parallel algorithms library to optimize block and thread counts. GPU threads synchronization is specifically designed to avoid data racing. Since all filopodia and fibers must interact in simulations, two 3-dimensional cell-grids of different resolutions are utilized to build nearest-neighbor maps. This grid method reduces the cost of element interaction from O(*n*^2^) to O(*n*), resulting in scalability and a nearly linear runtime increase with respect to element count. Additional comments on the computational cost of the model and GPU parallelization are available in Supplementary Note 1.4. Running sensitivity analysis requires hundreds to tens of thousands of model simulations (e.g., variance-based methods like Sobol’ indices; PRCC or eFAST^62,63^), which is currently computationally too expensive. Therefore, we restricted our analyses to those free parameters that we have identified as being most likely to have the largest biological impact. The results presented in the figures in the “Results” section were obtained by averaging measurements from 10 simulated clots. The figures plot both the mean and standard deviation of each output metric calculated from these simulations. We can see that the standard deviation is acceptable.

#### Comparison of our model with other models of clot contraction

We note that both our model and the models described in literature^20,51^ are developed for studying fibrin clot contraction and may seem similar. E.g., both models employ coarse-grained representation of fibrin fibers. However, the published models were designed to deal primarily with special aspects of clot contraction, platelet heterogeneity, and the relationship between forces generated by individual platelets and the bulk contractile force. Moreover, despite some apparent similarities, the models^20,51^ use dissipative particle dynamics to include the fluid effect (viscosity), while our model accounts for fiber–fluid interaction by introducing a viscous damping force, and thermal fluctuation of nodes by Brownian force. The models^20,51^ represent the elasticity of individual fibers as harmonic springs, while our model uses a nonlinear Worm-Like-Chain approach. Parameter values of the Worm-Like-Chain are obtained by fitting with measurements from atomic force microscopy experiments. Since a fibrin fiber exhibits nonlinear force-strain profiles, the nonlinear Worm-Like-Chain accurately represents the local strain–stress relation, which in turn affects the amount of force that a platelet can impose on a fiber. Models in literature^20,51^ apply volume exclusion energies to individual fiber nodes to prevent them from overlapping and do not form cohesion bonds between fibers after initialization of simulations. Our model dynamically forms cohesion bonds when two fibers are close to each other to prevent them from crossing each other. These models^20,51^ give each platelet exactly 12 filopodia, each pulling with a linear force on nearby fibers (i.e., force proportional to the clot contraction). Our model uses a probabilistic distribution for the number of filopodia determined using experimental data.

The local fiber densification by platelet pulling is modeled differently in these models. Our work captures densification by two proxies. First, is the overall time evolution of the area of platelet-colocalized fibrin, and second is the formation of platelet clusters which are observed to coincide with the local densification of fibrin. Other models^20,51^ measure the fiber network densification by proxy of volume and cross-sectional area. In simulations, another difference between the work^51^ and this paper is that we look at clot contraction and platelet clustering and determine the shrinkage of a clot in quasi-steady states; the iClot framework^51^ looks at the emergent macroscale force that their clot applies to boundary walls and the clot volume impact of temporal asynchrony of platelet contraction. The previous paper^20^ also looks at the cross-sectional area of clots and cross-link concentration (i.e., evolution of volumetric concentration of a static number of fibrin–fiber junctions) as a measurement of contraction. By contrast, we measure platelet clustering directly from simulation via individual platelet locations. Both papers^20,51^ represent systems with clots affixed to two fixed boundaries, whereas our simulations have free boundaries.

### Model validation

**Fig. 6:**
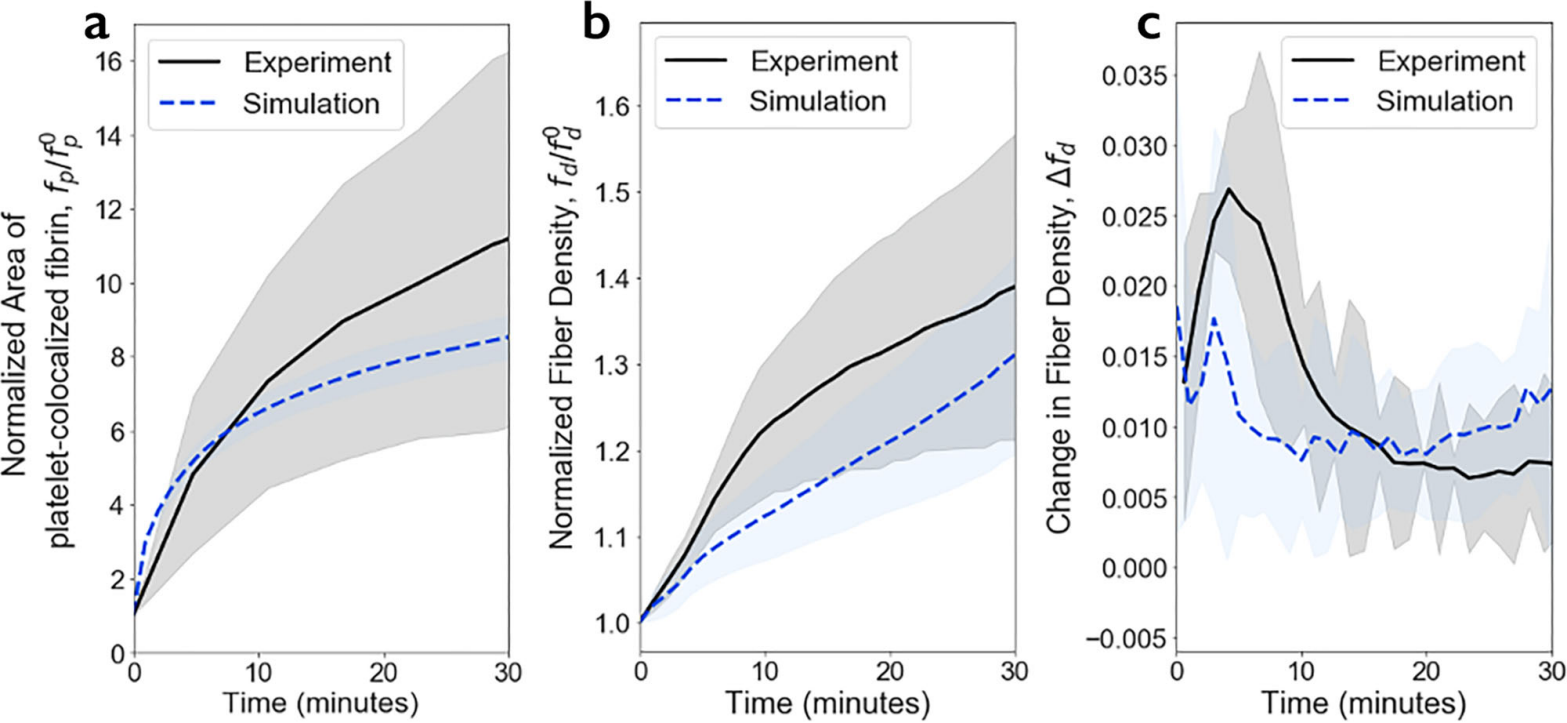
Platelet–fibrin co-localization and densification of fibrin networks during clot contraction. Simulation results are shown by blue dashed lines, while black solid lines represent experimental data^16^. Shaded regions represent the interval between mean plus one standard deviation and mean minus one standard deviation. **a** Normalized area of platelet-colocalized fibrin, ${f}_{{\rm {p}}}/{f}_{{\rm {p}}}^{0}$, during clot contraction. **b** Normalized fibrin density, ${f}_{{\rm {d}}}{/f}_{{\rm {d}}}^{0}$ as a function of time. **c** Changes in fibrin density, ${\varDelta f}_{{\rm {d}}}$ during clot contraction (the first derivative of ${f}_{{\rm {d}}}{/f}_{{\rm {d}}}^{0}$). Results are presented as *M* ± STD from 10 simulations (see also Supplementary Note 4.1 and Supplementary Fig. 4 for details on contraction phase calculations.

Simulations were performed for spherical clots with a radius of 23.8 µm, chosen to be biologically relevant and as large as practically possible to finish simulations in a reasonable time. As described in the “Methods” section, several forms of a filopod pulling forces were tested and the one best fitting experimental data was selected (see Supplementary Notes 3.2 and 4.3 for details). For each form of the force, we ran 10 clot contraction simulations with fixed parameter values indicated in Table 1 with independently generated initial structures of clots. Average and standard deviations for each contraction metric are provided in Fig. 6. These clot simulations involve 45 spherical platelets embedded into fibrin networks and they are run for 30 min of real contraction time. Each clot was generated computationally with an average platelet density of 800,000 platelets/mm^3^ corresponding to a platelet concentration in platelet-rich plasma (double the highest normal platelet count in whole blood to account for the volume of erythrocytes)^64^, and 0.3% fibrin volume fraction corresponding to a normal mass concentration of fibrinogen in plasma 0.2–0.4%^65^.

The fraction of fibrin initialized near platelets, ${P}_{{{\rm {fnp}}}}$, was fixed at 0.5 (see Supplementary Note 2.1), as this was the fraction corresponding to simulated networks being more qualitatively like the ones from experiments. Note that these fiber densities and platelet densities, within the chosen clot volume, result in over 60,000 possible attachment points (nodes and sub-nodes) for filopodia on fibrin fibers. The average initial inter-platelet distance in our simulations is 25 microns. Hence, platelet-platelet interactions are rare and only occur once two platelets are brought very close to each other during the network densification during clot contraction. The distribution of filopodia counts per platelet from four independent experiments imaging and analyzing more than 400 platelets are shown in Fig. 5 as light gray rectangles. The fitted lognormal distribution ($\mu =1.85$,$\sigma =0.27$), used in the simulations is displayed as dark gray rectangles. Simulated platelet filopodia counts used in simulations were obtained by sampling this lognormal distribution fitted using the experimentally observed distributions (as described in the “Methods” section).

The number of filopodia depends on the extent of platelet activation and cytoskeletal dynamics, irrespective of the amount of surrounding fibrin. The platelet–fibrin binding is probabilistic: the higher the fibrin density is and the more filopodia are formed, the more likely it is that they interact. The model implies that all filopodia are involved and this is a reasonable simplification. Moreover, since there is not much available data in terms of the precise distribution of which fibers platelets are attaching their filopodia, we allow them to choose randomly among fibers within their reach. Our model framework may be easily extended to accommodate alternative hypotheses for how platelets might choose which fibers to pull.

To validate the model, simulations of clot contraction were compared to experimental data from Kim et al. ^16^ using several appropriate metrics to quantify clot contraction over time (Fig. 6). Metrics used in Fig. 6a–c include the relative area of platelet-colocalized fibrin on the surface of the simulated platelets (i.e. the fraction of fibrin that lies within zones 2 or 3 of any platelet as described in Fig. 4) ${f}_{\rm {{p}}}$; fibrin fiber density, ${f}_{\rm {{d}}}$; and change in fibrin density, $\varDelta {f}_{\rm {{d}}}$. Figure 6 shows that simulated results are within a single standard deviation region of the experimental measurements. In particular, the model correctly predicts an increase in the amount of fibrin accumulated by individual platelets because of their pulling on fibrin fibers and fiber compaction (Fig. 6a). The calculated changes in fibrin density reveal a 1.4-fold increase after 30 min of contraction, which agrees with the experimental data (Fig. 6b).

These comparative results provide validation of the proposed model of clot contraction. In previous experimental studies^16^, three distinct kinetic phases were observed in clot contraction. These are “pre-contraction” or lag period (phase P1), “active contraction” (phase P2), and “final contraction” (phase P3). Changes in the first derivative of fibrin density function with respect to time indicate three kinetic phases in both in vitro and in silico contracting platelet-fibrin clots (see Fig. 6c and Supplementary Note 4.1).

The times of changes from one phase to another are identified by the two major peaks in the fibrin density changes (maxima in Supplementary Fig. 4) implying three kinetic phases of clot contraction, previously referred to as “pre-contraction”, “active”, and “final” contraction stages^16^. Remarkably, the kinetic phase borders for the model clot at *t* = 3.0 ± 1.0 min and *t* = 33.0 ± 1.0 min are very close to the temporal borders for experimental clots at *t* = 4.2 ± 1.2 min and *t* = 29.4 ± 1.2 min. The kinetic rates (i.e., average fibrin density change during a phase) of the three consequent contraction phases calculated in the model: *k*_*1*_ = 0.015/s, *k*_2_ = 0.010/s, and *k*_*3*_ = 0.011/s, are also partially in agreement with the corresponding experimental values *k*_1_ = 0.024/s, *k*_2_ = 0.011/s, *k*_3_ = 0.0044/s. In particular, the kinetic rate of the “active” phases for both experiments and simulations are almost identical.

To further validate the model, we ran experiments of a macroscopic platelet-rich-plasma clot contraction in the absence and presence of blebbistatin, an inhibitor of myosin IIA (see the “Methods” section), and compared them to the corresponding simulations, in which the effect of blebbistatin was mimicked by a 10-fold reduction of platelet pulling force. The experimental and simulated clot contraction kinetics curves are in quantitative and qualitative agreement (Supplementary Fig. 11).

**Fig. 7:**
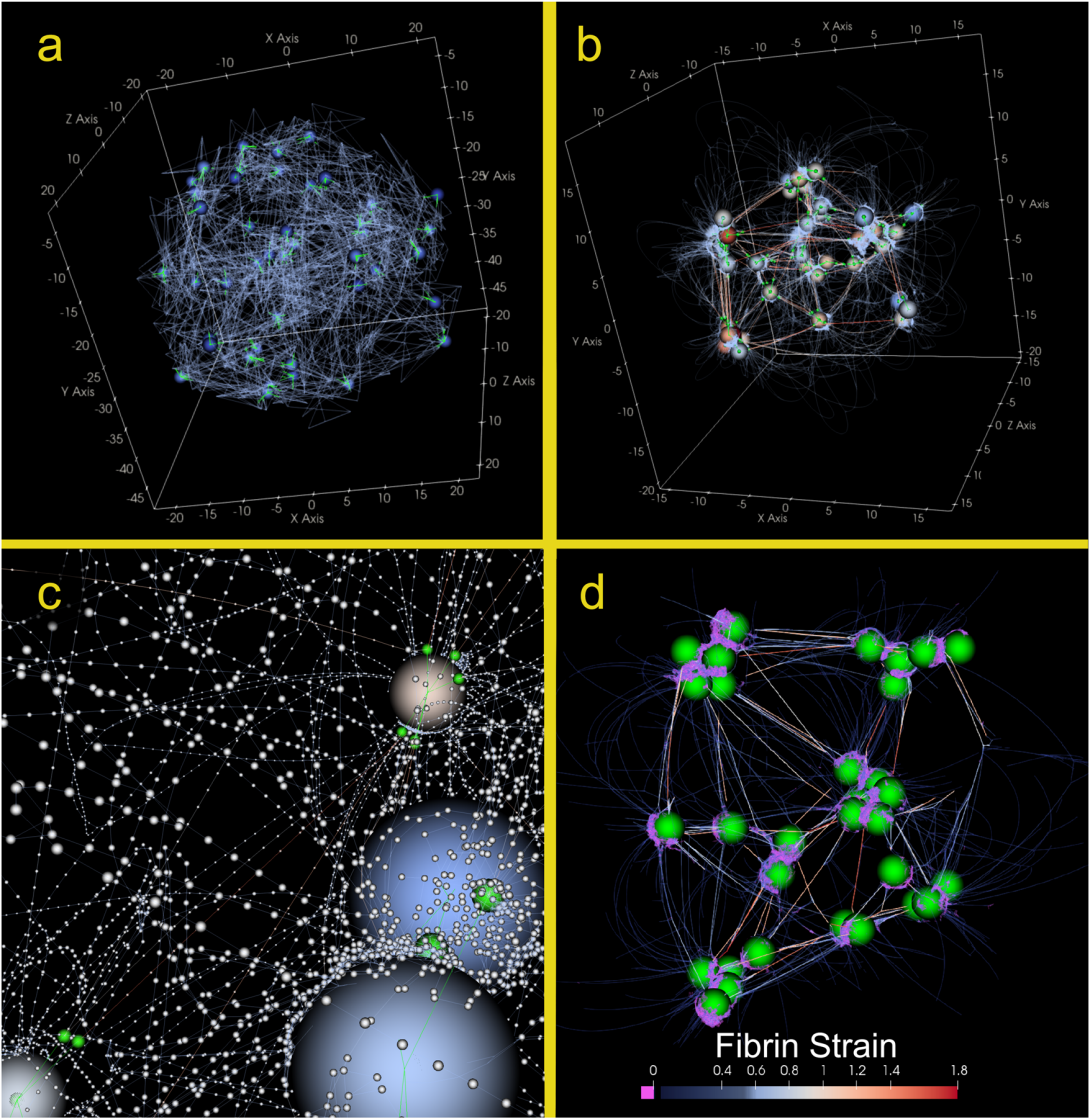
Images of simulated clots at different spatial scales. Simulated clot configuration at $t=0$ minutes (**a**) and *t* = 45 min (**b**), and a close-up of small-scale model elements (**c**). Each large sphere represents the body of a platelet inside a spherical clot of initial radius $25\,{\rm {\mu m}}$, comprising fibrin fibers (lines, and small white sub-nodes in (**c**)). The small green lines represent the filopodia, and the small green spheres show the point of active connection between filopodia and fibrin. Warmer-colored platelets are exerting more total $|{F}_{{{\rm {filopod}}}}|$ on their surrounding network. For better visibility, the individual fibrin fiber sub-nodes are not shown in (**a**) and (**b**), but lines drawn between them are colored by fiber strain from low strain (cooler colors) to higher strain (warmer colors). Moreover, to aid in viewing the platelet distribution, opacity of low-strain fibers has been reduced in (**a**–**d**). **b** shows the emergence of high-strain fiber bundles. Panel **c** is a close-up of several platelets within the simulated clot and depicts individual fibrin sub-nodes in white. This panel demonstrates fibrin accumulation on the body of the platelets. Panel **d** shows a 2D projected of a side-view of a clot that is digitally cut in half, showing the interior strain distribution as being primarily localized into bundles between platelet clusters, and compressed fibrin adherent to platelet walls.

Lastly, we noticed that in model simulations, after platelet clusters form, there is an emergence of high-strain fibrin bundles that form between platelet clusters having the spatial scale of tens of microns (Fig. 7, and Supplementary Movies 1–4) as observed before^66^. Supplementary Movies 1–4, Supplementary Note 4.5 and Supplementary Fig. 10 also demonstrate the localization of strain in bundles in model simulations.

### Model predictions of the impact of the number of platelet filopodia on the clot contraction kinetics

**Fig. 8:**
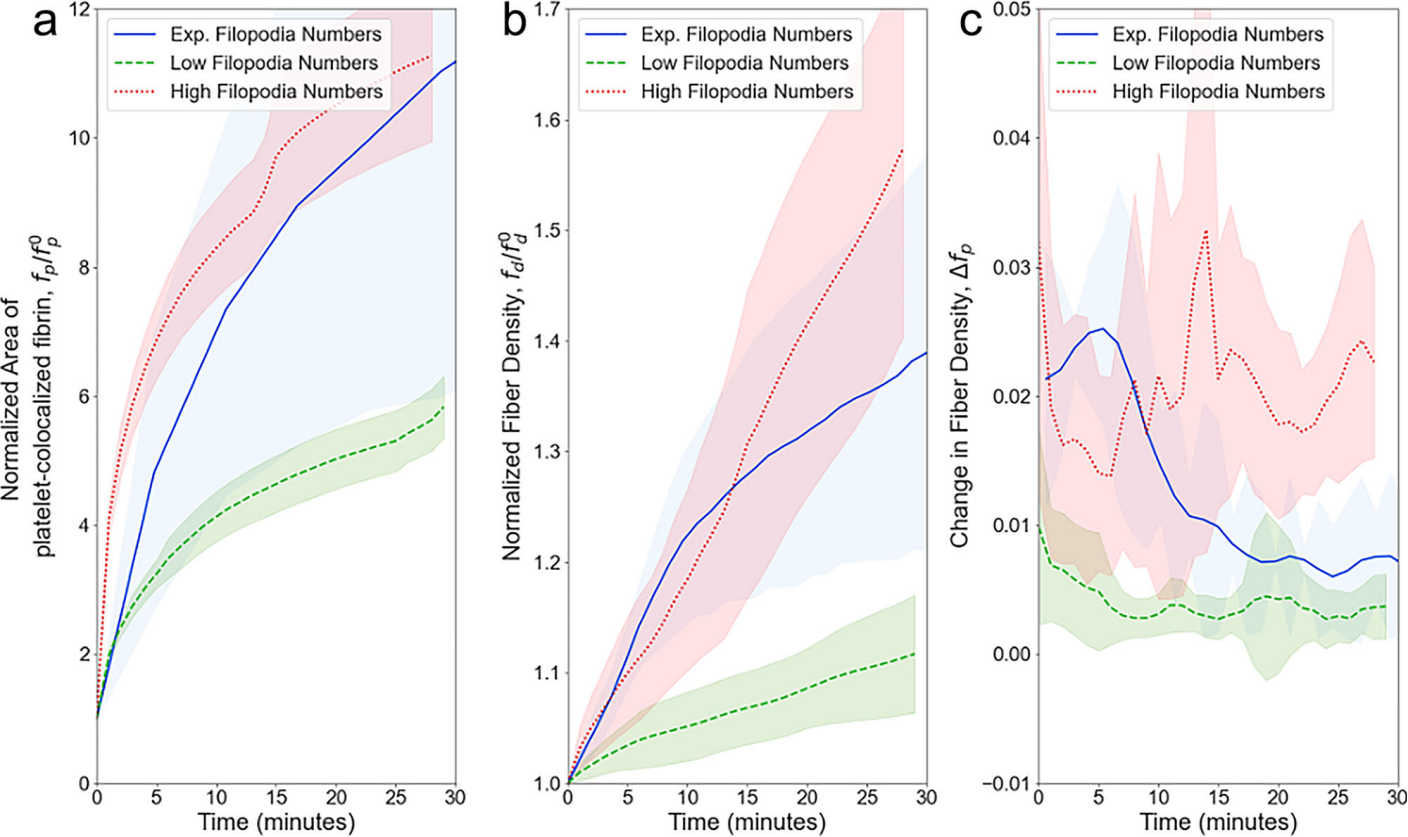
Simulated kinetics of clot contraction driven by platelets with different numbers of filopodia per 1 platelet. Simulations with experimentally observed intermediate filopodia numbers are shown by *blue solid lines*, while *green dashed lines* and *red dotted lines* represent simulations of clot contraction kinetics with the lower and higher filopodia numbers, respectively. Shaded regions represent the interval between mean plus one standard deviation and mean minus one standard deviation taken from *N* = 10 simulated clots. **a** Area of platelet-colocalized fibrin, **b** fiber density, and **c** changes in fiber density over time (see also Supplementary Note 4.2 and Supplementary Fig. 5 for details on contraction phases calculations).

Under different conditions, platelets have been shown to have various levels of activation, resulting in more or fewer filopodia per platelet^67,68^. The number of filopodia per platelet is one indicator of platelet activation in our model that we can easily control. To assess and quantify the impact of the number of individual filopodia, the clot contraction rate in addition to the metrics presented in the “Model validation” subsection, was calculated over 30 min of contraction in clots generated with varying numbers of filopodia per platelet. All other model parameters (Table 1) remained fixed. Clot contraction was simulated with the number of filopodia taken from the experimentally observed distribution (Fig. 5). The distribution with a lower number of filopodia corresponds to a lower degree of platelet activation. Conversely, the distribution with a higher number of filopodia corresponds to a higher degree of platelet activation^67,68^. Figure 8 shows the outcome values of the metrics presented in the previous section calculated from simulated clots for platelets with the experimentally observed distributions of filopodia numbers, and compared to the computationally derived filopodia numbers that are both lower and higher than the experimental values (Fig. 8a–c).

The rates and extents of clot contraction based on fibrin densification correlated with the degree of fibrin co-localization with platelets. In simulations, the fiber density is increased by approximately 10% in simulations with low filopodia numbers ($\mu =0.9$ and $\sigma =0.19$) and by more than 55% in simulations with high filopodia numbers ($\mu =3.7$ and $\sigma =0.38$), while platelets with the experimentally observed intermediate numbers of filopodia ($\mu =1.85$,$\sigma =0.27$) cause ~30% densification (Fig. 8b). Figure 8a shows that in the course of clot contraction, platelets with the experimentally observed intermediate filopodia numbers increase the amount of platelet-co-localized fibrin 8-fold, while platelets with low and high filopodia counts achieve a 5- and 10-fold increase in fibrin co-localization, respectively. Thus, the simulation results (Fig. 8) revealed that an increase in the number of filopodia per platelet resulted in faster clot contraction, more platelet-associated fibrin, and higher fiber density.

Remarkably, the simulated clots containing platelets with the experimentally observed filopodia counts had absolute contraction metrics values closer to the ones observed in experiments^16^ (see also Fig. 6), while the simulated clots with lower or higher numbers of filopodia per platelet underwent reduced or enhanced contraction, respectively, as can be seen in Fig. 8. This correlation provides another argument for the experimental validation of the model applied to simulate the kinetics of clot contraction.

Thus, model simulations suggest that the number of contracting filopodia per platelet is an important characteristic that determines the phase kinetics of clot contraction and its biomechanical complexity. Platelets with a reduced number of contractile filopodia yield impaired clot contraction with a single kinetic phase, while an increase in the number of contractile filopodia per platelet leads to clot contraction characterized by three or more kinetic phases, reflecting a more complex biomechanical mechanism of blood clot contraction when platelets are more activated.

### Model prediction of platelet clustering during clot contraction

**Fig. 9:**
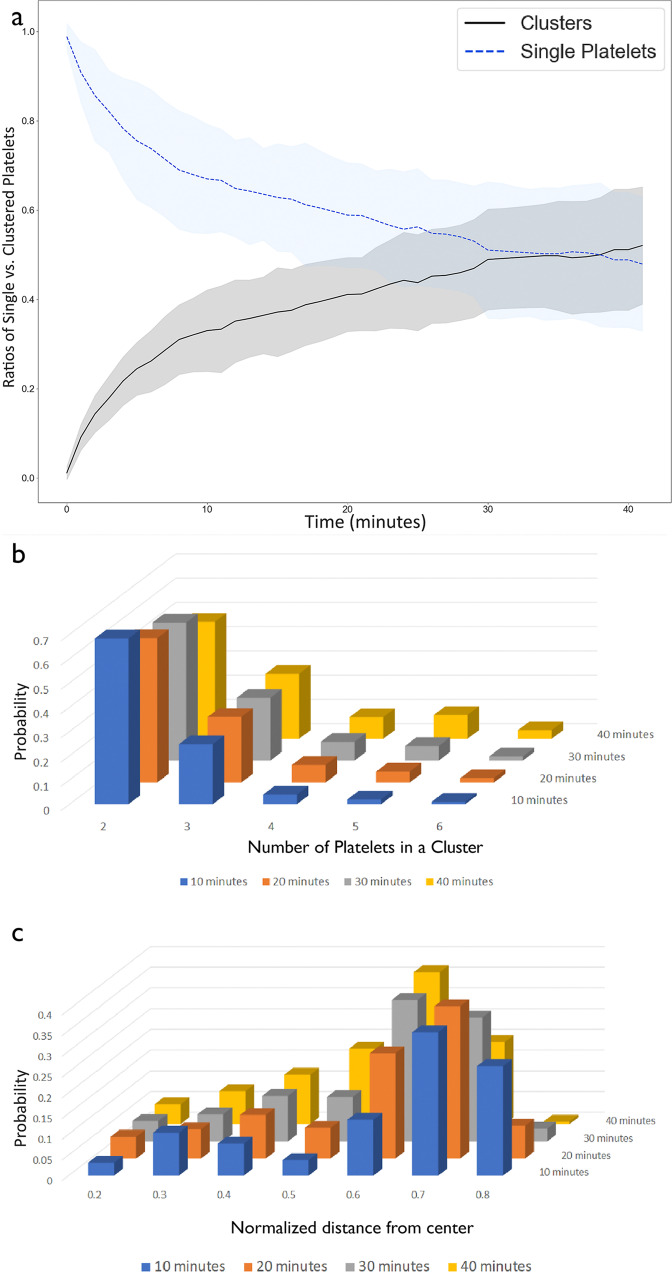
Platelet clusterization during clot contraction. **a** Ratios of the numbers of single platelets (*dashed blue line*) and platelets in clusters (2 or more platelets, *black solid line*) during clot contraction. Solid lines represent mean values, shaded areas indicate standard deviation from the mean, *N* = 10 simulations. **b** Histograms of the number of platelets per cluster, containing 2–6 platelets formed in simulated clots over the course of contraction. Time points: 10 (*blue*), 20 (*orange*), 30 (*gray*), and 40 (*yellow*) minutes. **c** Histogram of the distribution of platelet clusters at various normalized distances from the center (normalized with respect to the radius of clot before contraction) observed in the simulation. Time points: 10 (*blue*), 20 (*orange*), 30 (*gray*), and 40 (*yellow*) minutes.

It has been previously shown^16^ that the inter-platelet distances in contracting clots progressively reduce throughout contraction, which can result in the formation of secondary platelet clusters that can potentially reinforce the contraction. Model simulations also demonstrated the ability of platelets to clusterize during fibrin clot contraction. Single platelets were initially uniformly dispersed in a clot with an average inter-platelet distance of 25 μm. As a result of fibrin network densification during clot contraction, platelets formed clusters consisting of two or more cells (Fig. 9a). In 10 min of clot contraction, most platelets were twinned and later the number of clusters, and their size progressively increased. Between 10 and 30 min, the average fraction of two-platelet clusters decreased, while the fraction of clusters containing three, four, five, and six platelets increased by 30%, 160%, 250%, and 100%, respectively (Fig. 9b). Around 40 min after initiation of clot contraction, the number of clustered platelets surpassed the fraction of single platelets in the clot (Fig. 9a).

Figure 9c represents the frequency of platelet clusters with respect to their distance from the clot center at times *t* = 10, 20, 30, and 40 min from the beginning of clot contraction. Therefore, our simulations predict that clusters first form closer to the edge of the clot. In particular, platelet clusters are more frequent in domains further from the center and closer to the boundary of the clot (Supplementary Note 4.4 and Supplementary Figs. 7–9 for additional details). As clot contraction progresses, the cluster positions shift closer to the core of the clot (Fig. 9c), reflecting the macroscopic shrinkage of the clot from the edge toward the center. This simulation result agrees with the contraction front propagation from the boundary to the center of the clot observed experimentally^16,51^.

## Discussion

Multiscale computational models describing interactions between cells and extracellular matrices and calibrated using specific experimental data play an important role in studying the biomechanics of cells and tissues, as well as embryo development and cancer invasion^69–72^. In particular, several theoretical and computational models have been developed to simulate the properties of fibrin and the structural mechanics of fibrin networks at different spatial scales^28,30,73–85^. Detailed computational mechanical models of platelets and red blood cells were described in refs. ^86,87^. Also, the stability of a clot under different flow conditions using multi-phase models was previously studied in refs. ^84,88–90^.

While blood clot formation has been relatively well studied, little is known about the mechanisms underlying the subsequent complex processes of structural and mechanical clot remodeling called *contraction* or *retraction* which are of high pathophysiological significance. Clot contraction is driven by activated platelets that pull on fibrin fibers, causing a reduction in clot volume. The results obtained by Kim et al. ^16^ established a quantitative structural basis for the mechanobiology of blood clot contraction. Human platelet-rich plasma clots were experimentally examined at a variety of spatial scales, with a particular focus on single platelet–single fibrin fiber interactions, and their effects on the kinetics of clot contraction at the macroscopic level. In this paper, experimental observations were complemented and advanced by multiscale computational modeling to provide important mechanistic insights into the contraction and structural remodeling of blood clots.

The multiscale modeling framework for simulating clot contraction, described in Fig. 2, combines a microscale sub-model of the “pulling hand-over-hand on a rope” mechanism of a filopod pulling on a fibrin fiber based on the fiber’s stiffness with a mesoscale sub-model of the fibrin network. The model was validated using specific experimental data by demonstrating similar kinetic rates of the “active” phases of contraction for both experiments and simulations. Then, model simulations successfully quantified the fibrin compaction/accumulation near individual platelets and platelet aggregates. Also, in this paper, we varied the number of filopodia per platelet and predicted how an increased or decreased number of filopodia per platelet could influence the contraction process. Moreover, model simulations showed that most of the mechanical strain within a contracting clot was experienced by bundles of fibrin fibers spanning between individual platelets and/or their clusters.

To correctly account for the rate of a single filopod retraction, we note that the intra-platelet actomyosin machinery generating the traction forces is energy-demanding and is strongly ATP-dependent. With the consumption of the intracellular pool of ATP, the ability of the platelet to contract decreases over time. Natural platelet exhaustion results in a gradual decrease and abolition of platelet contractility^7^. Another mechanism that restricts clot contraction is gradual stiffening of the deformed fibrin which makes contracting fibrin progressively resistant to platelet-induced deformations^91^. Both energetic exhaustion and fibrin stiffening explain the plateau in the fibrin network densification. The model implicitly accounts for the rate of filopodia retraction by considering the force generated by platelet filopodia, which in turn depends on fibrin stiffness.

The reason for impaired clot contraction in (pro)thrombotic conditions is platelet dysfunction and exhaustion, which is secondary to continuous platelet activation^3,4^. One of the main consequences of such exhaustion is platelet refractoriness, i.e., the reduced ability to respond to an activating stimulus. The model captures this situation by studying platelets with various degrees of activation, including the reduced ability to form filopodia, which corresponds to partial platelet refractoriness.

Because the degree of platelet activation correlates directly with the number of platelet filopodia, our findings suggest that weak platelet activation results in impaired clot contraction. This is consistent with previous in vitro data showing that the decrease in thrombin concentration from 1 to 0.5 U/ml, and hence the extent of platelet activation, reduces the degree and rate of clot contraction^41^. The dynamic relation between agonist concentration in a clot and the number of filopodia generated by a single platelet is currently not known. Perhaps if such a relation is developed in the future, our model can be extended accordingly.

Model simulations with an average number of 6–7 filopodia per platelet reproduced the three contraction phases observed in Kim et al. ^16^ with similar duration and fibrin densification rates. Additionally, model simulations predict that an increase in the number of filopodia to 12 results in 4 phases, with higher fibrin densification rates. On the other hand, a decrease in the number of filopodia to 2–3 produced only a single phase, with a lower fibrin densification rate. Change in the number of phases means a change in contraction kinetic rates. As impaired clot contraction has been associated with postoperative deep vein thrombosis^4^, our results complement these findings suggesting that fibrin densification in clots of these DVT patients could be significantly reduced. Meanwhile, the number of kinetic phases in DVT patients was unaltered in comparison to healthy control, pointing out the role of platelet exhaustion in impaired clot contraction.

Model simulations also quantified the potential impacts of different levels of dependence of filopodia pulling forces on the stiffness of the fibers, the loading time of these forces, as well as competition for actomyosin between filopodia of the same platelet (see Fig. 2, Supplementary Note 4.3, Supplementary Table 1, and Supplementary Fig. 6), on the rate of change of fibrin density of a clot during contraction. It was also demonstrated by tracking platelet clusterization in simulations that the formation of platelet clusters was more prominent at the edges at the beginning of the contraction and subsequently, these clusters moved toward the center. Mechanistically, platelets at the edge of a clot experience larger net radial force, than platelets located closer to the clot center. As a result, the radial velocity of platelets at the edge of the clot is higher than that of platelets closer to the clot center. This radial velocity gradient for platelets leads to their clustering at the clot edge, as platelets from the edge overtake platelets located closer to the clot center over the course of contraction.

In this study, 50 μl of human plasma was used to generate an mm-size clot to study its contraction in vitro. 120 × 120 × 35 μm confocal microscopy z-stack images of clots were acquired during clot contraction and used for structural analysis. By running simulations for incrementally increasing initial clot size, and calculating the final extent of contraction, we found that the results did not significantly change as the initial clot radius reached ~20 μm, suggesting that the model is rather scalable, and results are applicable to macro-scale clots. Since the model’s complexity scales linearly with the volume of the simulated clot, simulations of larger clots are possible once the architecture for multi-GPU simulations has been built. This simulation framework is under development and may be used in the future to perform in-silico studies on much larger-order clots.

The multiscale modeling approach described in this paper can be used to further explore properties of the contraction process that could not be easily measured in experiments (see Fig. 2). Some of these properties are known to either be dependent on the location where the clots formed (e.g. different fiber orientations in different types of blood vessels, depending on flow rates) or alterations in patients with blood pathologies, such as Bernard–Soulier syndrome, characterized by mutations in the GPIb-IX-V complex, accompanied by unusually large platelets, low platelet counts and prolonged bleeding time, or some congenital dysfibrinogenemias characterized by fibrinogen mutations that result in alterations in fibrin structure and properties. Provided the right data to estimate the parameter values, the multiscale model can reproduce clots formed in any of these alternative scenarios and the results of simulations can be used to predict clot contraction. This could be especially useful to devise possible drug treatment strategies for some of the bleeding or thrombotic disorders that affect clot contraction.

We note that the current model does not include RBCs which are known to play an important role in blood clot formation and contraction^7,42^. Clot contraction results in the deformation of RBCs into tightly packed polyhedral-shaped cells, polyhedrocytes or piezocytes, forming an impermeable barrier important for hemostasis. Since the goal of this paper is to evaluate the mechanism of platelets pulling on the fiber network, we excluded RBCs from our experiments and simulations to be able to clearly test platelet–fibrin adhesion and mechanical interactions. The interaction between RBCs and fibrin fibers is passive in the sense that there is no measurable inherent contractile force exerted by RBCs on fibrin. We plan to extend our multiscale model in the future to include RBC representation, like the ones from literature^92–95^, as well as our extended multiphase submodel^96^ to study the contraction of blood clots with multiple types of cells.

It is well known that fibrin fiber networks formed under flow conditions^97^ have a structure different from that of ones formed under static conditions. However, fiber networks formed under static conditions in vitro and flow conditions in vivo have similar mechanisms and comparable extents of clot contraction^98,99^. Since the experiments that we used in this work are performed under static conditions (the Reynolds number is $\ll$1), we, therefore, do not explicitly include fluid flow equations in our model. Nevertheless, we account for fibrin fluid interaction within the clot by introducing a viscous damping force, and thermal fluctuation of nodes by Brownian force. We also note that the values of parameters in the current model are independent of blood flow. Moving forward, further model developments will involve the placement and stiffness of RBCs localized in different regions of the clot before contraction to see its impact on clot shape and size; such impacts are evidently shown by works such as^100^ to be important. Such simulations may provide insights into the impact of pathological stiffness of RBCs on blood clot contraction^101^, which would be difficult to study in vivo or to design costly experiments more efficiently. Alternatively, we will extend our fibrin-blood flow interaction equations to include a biased drift force proportional to the fibrin’s proximity to the clot surface, as observed in literature^39,96^. Studies on the impacts of blood flow on contraction are difficult and costly in animals, and not possible in human subjects.

Finally, the mechanisms and computational modeling approach described in this paper could also apply to other systems in which cells or particles are embedded in a fiber network and exert forces on their surroundings based on substrate stiffness or other mechanical properties. For instance, fibroblasts reorganize the fiber networks composed of collagen and elastin in asthmatic and healthy airways (i.e., organs of the respiratory tract that allow airflow during ventilation). Another example is bacteria interacting with a mycotic network, hence acting similarly to platelets in the fibrin network.

## Methods

### Formation and contraction of platelet-rich plasma clots

Human blood was drawn by venipuncture from healthy volunteers not taking aspirin or other medications affecting platelet function for at least 14 days. Informed consent was obtained following a protocol approved by the University of Pennsylvania Institutional Review Board. Platelet-rich plasma (PRP) was prepared from whole blood drawn into 3.8% trisodium citrate (9:1 v/v) by centrifugation at 210×*g* at 25 °C for 15 min. To label fibrin and platelets, the PRP samples were pre-incubated with Alexa-594-labeled human fibrinogen (9 µg/ml) and calcein (10 µg/ml), respectively, for 10 min at 37 °C. To induce clotting and clot contraction, PRP samples were re-calcified with CaCl_2_ (29 mM) and mixed with human α-thrombin (1 U/ml, all final concentrations). An activated plasma sample was immediately transferred onto a microscope glass surface of a PELCO cell culture dish inside the environmental chamber of a confocal microscope. The glass surface was pre-coated with 4% (v/v) Triton X-100 to prevent attachment of fibrin to glass and allow for unconstrained clot contraction.

### Confocal microscopy and image analysis of contracting clots

PRP clots were imaged using a Zeiss LSM710 laser scanning confocal microscope with Plan Apo ×40 (NA1.2) water immersion lens to acquire serial 35 μm-thick z-stack images of the clots during contraction (40 min, at time intervals of 75 s). The distance between slices of the z-stack images was 0.8 μm; each image was taken at a 1024 × 1024 pixels resolution. Fluorescently labeled fibrin and platelets were excited using 594-nm helium–neon (fibrin) and 488-nm argon (platelets) laser beams and visualized at 620 nm (red) and 515 nm (green) emission wavelengths, respectively. 3D reconstruction of the fibrin-platelet mesh was done using Imaris software. The number of filopodia formed by individual platelets was measured manually in the acquired confocal microscopy z-stacks of contracting PRP clots using the open-source Fiji software. To calculate the number of filopodia, each image was divided into 50 square domains of 10-µm-thick z-stacks, and the number of filopodia for each individual platelet was counted. To track changes in fibrin fluorescence intensity, density of the fibrin network, and colocalization of platelets with fibrin, serial confocal microscopy z-stack images collected during clot contraction were post-processed using Fiji software and quantified using standard Fiji image analysis tools^16,102,103^.

### Clot contraction assay

Platelet-rich plasma (PRP) was obtained by centrifugation of citrated whole blood at 200 g for 10 min at room temperature. PRP samples were preincubated without and with blebbistatin (200 µM final concentration) for 3 min at 37 °C. Clot size changes were tracked optically over time as described previously^41^. Plasma clots were formed in plastic cuvettes whose walls were pre-lubricated with 1% pluronic F-127 (Sigma-Aldrich) to prevent fibrin from sticking. To initiate clot formation, in a separate plastic tube citrated PRP was recalcified with 2 mM CaCl_2_ followed by the addition of 1 U/mL thrombin (both final concentrations). For clot formation and contraction, 80-μL samples were transferred to transparent plastic cuvettes that were imaged using an optical instrument equipped with a CCD camera. Changes in clot size during contraction were followed by acquiring images every 30 s for 30 min. Image sequences were analyzed computationally to plot contraction kinetic curves and measure the final extent of contraction.

#### Generation algorithm of the initial fibrin network and platelet structures: input parameter values used for simulation

The values of the parameters used in the model are given in Table 1. To obtain a virtual fibrin network with properties both quantitatively and qualitatively similar to those observed in the experimental platelet-rich plasma clots, we developed an algorithm to generate a computational three-dimensional clot consisting of both platelets and fibrin fibers. This routine uses quantitative measures extracted from confocal microscopy imaging of pure fibrin^27,29^ or plasma clots^27,29^, such as fiber length distribution, fiber orientation, and fiber densities. These quantities and the structures of the clots are fundamental for the proper biologically relevant contraction dynamics in blood clots. (The algorithm and details of its application are described in Supplementary Note 2.1.)

#### Statistics and reproducibility

Simulations of 10 independently generated in silico clots composed of fibrin and activated platelets were performed and statistically analyzed. This was done for model validation and model prediction verification. We utilized 3-degree polynomial curves for fitting different data sets in Supplementary Note 3 by performing a non-linear least-squares optimization routine from the SciPy Python library^104^. The errors (indicated by shaded segments, see Figs. 7 and 8 in the main text and Supplementary Figs. 4–6) were calculated using standard deviations in each fitting polynomial coefficient given by ${\sigma }_{i}=\sqrt{{{\rm {co}{v}}}_{{ii}}}$ where ${\rm {{co}{v}}}_{{ii}}$ is the diagonal element of the calculated covariance matrix of the optimized values of the estimated coefficients.

### Reporting summary

Further information on research design is available in the Nature Portfolio Reporting Summary linked to this article.

### Supplementary information

Supplementary Material

Supplementary Movie 1

Supplementary Movie 2

Supplementary Movie 3

Supplementary Movie 4

Reporting Summary

## Data Availability

All relevant supporting data are available in the following CodeOcean repository: https://codeocean.com/capsule/4c7bb411-62de-47de-93b4-2c03441d6338/. The data for this study was generated with the simulation algorithms described in the paper and source code deposited in the CodeOcean capsule. Instructions for compiling the source code, running simulations to reproduce the results presented in the paper are in the README file.

## Code availability

Source code, input scripts, post-processing python files, and README files are available in the CodeOcean capsule: https://codeocean.com/capsule/4c7bb411-62de-47de-93b4-2c03441d6338/.
