# Supplementary material for: Combined computational modeling and experimental study of the biomechanical mechanisms of platelet-driven contraction of fibrin clots
Source: Commun Biol. 2023 Aug 24;6:869. doi: 10.1038/s42003-023-05240-z (PMC10449797; doi:10.1038/s42003-023-05240-z)
Supplement: Supplementary file 1 — Supplementary Material [file 42003_2023_5240_MOESM1_ESM.pdf]

Supplementary Material

Combined computational modeling and experimental study of the biomechanical mechanisms of platelet-driven contraction of fibrin clots

Christian Michael[1,2,3, #], Francesco Pancaldi[1,2, #], Samuel Britton[1,2], Oleg V. Kim[4,5], Alina D. Peshkova[6], Khoi Vo[1,2], Zhiliang Xu[7], Rustem I. Litvinov[4], John W. Weisel[4, *], Mark Alber[1,2,8 *]

[1]Department of Mathematics, University of California Riverside, Riverside, CA, 92505, USA

[2]Center for Quantitative Modeling in Biology, University of California Riverside, Riverside, CA, 92505, USA

[3]Department of Microbiology and Immunology, University of Michigan, Ann Arbor, MI, 48109, USA

[4]Department of Cell and Developmental Biology, University of Pennsylvania School of Medicine, Philadelphia, PA, 19104, USA

[5]Department of Biomedical Engineering and Mechanics, Center for Soft Matter and Biological Physics, Virginia Tech, Blacksburg, VA 24061, USA

[6]Department of Pharmacology, University of Pennsylvania School of Medicine, Philadelphia, PA, 19104, USA

[7]Department of Applied and Computational Mathematics and Statistics, University of Notre Dame, Notre Dame, IN, 46556, USA

[8]Department of Bioengineering, University of California Riverside, Riverside, CA, 92505, USA

Supplementary Note 1 - Model description

In this section we provide some additional details about several aspects of our multiscale computational model. Also, **Supplementary Table 1** includes a list of processes and space and time scales represented in the multiscale model.

The model requires the use of several computational techniques to avoid unreasonable runtimes. First, all independent operations are performed in parallel using the Thrust parallel algorithms library to optimize block and thread counts. Force calculations for individual platelet filopodia, fiber nodes, and platelet-fiber interactions are performed on Nvidia GPUs with synchronization before and after solving the respective ordinary differential equations in time. Second, since all filopodia and fibers must interact, two 3-dimensional cell-grids of different resolutions are utilized to build nearest-neighbor maps.

Supplementary Table 1: Clot contraction processes and scales

Processes	Spatial and temporal scales
Microscale: Fibrin accumulation near platelets	Spatial: from a few to tens of μm (depending on platelets cluster size)<br>Temporal: from a few minutes to a few hours (30 minutes in the model simulations)
Microscale: Fibrin-platelet interactions	Spatial: from less than 1 μm to a few μm<br>Timescale: from a few seconds to a few minutes
Mesoscale: Platelet clustering	Spatial: from few to tens of μm (depending on platelet cluster size)<br>Temporal: from a few minutes to a few hours (30 minutes in the model simulations)
Macroscale: Fibrin network contraction	Spatial: from tens of microns to mm (47.6 μm maximum clot diameter in our simulations)<br>Temporal: from a few minutes to an hour (30 minutes in the model simulations)
Macroscale: Contraction of a whole clot	Spatial: from tens of microns to mm (47.6 μm maximum clot diameter in the model simulations)<br>Temporal: from a few minutes to an hour (30 minutes in the model simulations)

The grid for the network has a lattice size given by $10 \cdot\ 2r_{fib} = 1\mu m$ while the grid for the platelets has a lattice size of $1.5 \cdot r_{fil} = 3.3\mu m$. This grid method reduces the cost of element interaction from $O(n^2)$ to $O(n)$, resulting in scalability and nearly linear runtime increase with respect to element count. In our computational model, both main components (fibrin network mechanics and platelet mechanics) act on mechanical properties and generate mechanical forces.

At each time step the neighbor list for each fibrin (node or sub-node) and for platelets is built using the grids described above. Only the nodes in the same neighborhood are considered when calculating interactions. This is done to reduce the computational cost while maintaining high computational accuracy. The forces acting on each node (fibrin node or sub-node or platelet) are then calculated. In particular, at each time step the strains of all fiber sub-segments are calculated and used to determine the elastic forces, between fibrin nodes and sub-nodes, and to determine the pulling force of each currently pulling filopodia as their force depends on the strain of the sub-segments connected to the node (or sub-node) that is currently being pulled. Then a forward Euler method is used to solve the system of differential equations for fibrin fibers and platelets nodes. Finally, the process is repeated for the next time step until the prescribed computational time is exhausted.

Supplementary Note 1.1 - Individual platelet and fibrin fiber sub-models. Platelets and fibrin dynamics are described by Langevin equations as follows. Motion of the location of each platelet center of mass is represented by a solution of the stochastic ordinary differential equation

$$m_{p,i}\ddot{x}_i = F_{p,i} - \eta_p \dot{x}_i + F_i^B \quad (1)$$

where $F_{p,i}$ is the deterministic force acting on the i-th platelet, $\eta_p \dot{x}_i$ is the viscous damping force, and F_i^B is the Brownian force due to thermal fluctuations [1]. We consider the system to be in an overdamped regime as in Britton et al. [2], due to the small Reynolds number. The inertial term is therefore neglected, and the system (1) is discretized in time using a Forward Euler scheme:

$$x_i^{n+1} = x_i^n + \frac{dt}{\eta_p}\left(F_{p,i}^n + F_i^{n,B}\right) \quad (2)$$

where the superscripts *n* and *n*+1 refer to the vector quantitates at time steps *n* and *n+1* for the i-th node and $dt = 0.001s$ is the time step. η_p represents the drag coefficient for a platelet with fixed volume and it is calculated using the Stokes estimation, $\eta_p = 6\pi\mu r_p$ [3]. The blood viscosity is $\mu = 0.004\, Pa.s$ and the platelet radius is $r_p = 1.13\, \mu m$, which results in the value $\eta_p = 85.2\, nN.s.m^{-1}$. Similarly, dynamics of fiber nodes is determined by solution of a Langevin equation

$$m_{f,j}\ddot{x}_j = F_{f,j} - \eta_f \dot{x}_j + F_j^B \quad (3)$$

where $F_{f,j}$ is the deterministic force acting on the j-th fiber node, and η_f is the drag coefficient for a fiber node with mass $m_{f,j}$. The system is discretized and solved in time using the same scheme:

$$x_j^{n+1} = x_j^n + \frac{dt}{\eta_f}\left(F_{f,j}^n + F_j^{n,B}\right). \quad (4)$$

Assuming fibers have a diameter of 100 nm, the value of η_f was calculated using $r_p = 0.05\, \mu m$ to be $\eta_f = 3.77\, nN.s.m^{-1}$.

The Brownian force in three dimensions is calculated using the Einstein relation between the diffusion coefficient of a medium and the temperature [4]. Because the mean squared displacement of a particle following Brownian dynamics is calculated as $< x^2 > = \frac{6k_bT}{\eta}dt$ over a single time step, the random force acting on a fiber node is $F_i^{n,B} = \xi_i\sqrt{\frac{6k_bT}{\eta_f}dt}$, where ξ_i is sampled from a standard normal distribution. Random forces are calculated similarly to the case of platelets with the drag term, η_p. The deterministic forces, $F_{p,i}$ and $F_{f,j}$, acting on each platelet's center of mass or fiber node, are separated into components as follows. For each platelet, the forces are generated by (1) pulling forces of filopodia, (2) adhesive forces and (3) volume exclusion forces. The total force is calculated using the negative gradient of the

energy, $F_i = -\nabla E_i^{PLT}$, where E_i^{PLT} is the energy associated with the i-th platelet separated into three components:

$$E_i^{PLT} = E_i^{FIL} + E_i^{ADH} + E_i^{EXC} \quad (5)$$

with E_i^{FIL}, E_i^{ADH}, and E_i^{EXC} representing the filopodia, adhesion, and volume exclusion energies determined as follows:

$$-\nabla E_i^{FIL} = -F_{platelet} \cdot \frac{x_i - x_j}{|x_i - x_j|} \quad (6)$$

$$E_i^{ADH} = F_0 \frac{1}{2}(x)^2 \quad (7)$$

$$E_i^{EXC} = \epsilon\left(\frac{\sigma^{12}}{x^{12}} - 2\frac{\sigma^6}{x^6}\right) \quad (8)$$

where $x = x_i - x_j$ is the distance between the i-th platelet and the j-th platelet/fiber, $\sigma = 2f_r r_p/2^{1/6}$, and $\epsilon = {F_0}^2 \cdot 1\mu m/nN = 16nN \cdot \mu m$. Note that in the main text that we refer to $|-\nabla E^{FIL}| = F_{platelet}$, $-\nabla E^{ADH} = F_0$, and $-\nabla E^{EXC} = F_{LJ}$. Since we did not have specific data to calibrate the Lennard-Jones forces, we have chosen parameter values based on computational stability. The minimum of the Lennard-Jones potential (see formula 8) is achieved when $\sigma = x$. Our choice of parameter values places the minima at $x = \sigma = 2f_r r_p/2^6 < 2f_r r_p$ assuring that the minimum of Lennard-Jones energy is achieved slightly inside the incompressible bodies of two neighboring platelets (since platelet bodies can partially deform when they aggregate). The choice of $\epsilon = {F_0}^2$ was determined by the assumption of Lennard-Jones forces being stronger than the adhesive force $F_0$and computations showed stable results using $\epsilon = {F_0}^2$. Similarly, the force acting on each fiber is calculated using the same formula (5) but with the force term separated into (i) forces applied externally to the network by platelets which generate (ii) individual fiber forces and (iii) bending forces. Each individual fiber is represented by a nonlinear Worm-Like-Chain (WLC) spring and by bending springs introduced at each node triplet as in Britton et al [2]. Thus, the total deterministic energy of the j-th fiber is as follows:

$$E_j^{FIB} = E_j^{PLT} + E_j^{WLC} + E_j^{BEND} \quad (9)$$

where E_j^{EXT}, E_j^{WLC}, and E_j^{BEND} represent the fiber, WLC and bending energies, respectively:

$$E_j^{WLC} = \sum_k \frac{Nk_bTC}{4P}\frac{3\bar{x}^2 - 2\bar{x}^3}{1-\bar{x}}, \quad \bar{x} = \frac{x_j - x_k}{C} \quad (10)$$

$$E_j^{BEND} = \sum_{n \in V(j)} \frac{B_s}{2}\left(\theta_{ljk} - \theta_0\right)^2 \quad (11)$$

where k_b is the Boltzmann constant, T is the absolute temperature, C and P are the scaled persistence and contour lengths. The normalized strain, $\bar{x}$ is calculated between the j-th and k-th nodes [5]. Each spring is represented using N for the number of fibrin monomers in parallel, and the normalized contour length C representing the number of fibrin monomers in series [5]. The energy of WLC springs acting on node j is calculated by summing up impacts from neighboring nodes. The energy on the node j is calculated by summing over all triplets, $V(j)$, surrounding it. Bending energy is equal to the bending constant, B_s, multiplied by the change between the current angle θ_{ijk} and the preferred angle θ_0 between nodes i, j, and k (see also **Figure 3** in the main text).

Formation of cohesion bonds leading to fiber crisscrossing in our model, instead of the volume exclusion principle, prevents fibrin fibers from passing through each other. Specifically, at each time step, distances between initially disconnected fiber nodes are compared with a threshold. If the distance between nodes of different fibers is less than the fiber diameter of 0.1 μm, a flexible link is established between the nodes to represent a fiber-fiber cohesive bond (See also Britton et al. [2].)

Moreover, as the platelet's filopodia pull fibers towards the platelet body, fibrin accumulates on the surface of the platelet. Volume exclusion between platelet and fibrin fibers is implicitly represented in the model as follows. Once the surface of the platelet gets saturated by fibrin as a result of filopodia pulling on fibrin fibers, any nodes representing additional fibrin fibers near platelet surface will be repelled by the WLC forces. This repulsion eventually balances with the filopods' pulling forces, leaving the clot at rest. This accumulation of fibrin on the platelets surface can be seen in **Figure 7**.

Supplementary Note 1.2 - Platelet-platelet interactions. Two platelets can interact if the distance between their centers of mass d_{pp} satisfies $2r_p < d_{pp} < 2(r_p + r_{fil})$ (i.e. the respective regions 1 of the two platelets overlap but do not overlap with region 2 or 3 (see **Figure 4** in the main text). If the filopod attaches to another platelet, when $2r_p < d_{pp} < 2(r_p + r_{fil})$, the force $F_p(t) = f_0/2$, the minimum force exerted by a filopod (see formula (5) in the main text). This model assumption was chosen so that if two platelets are pulling on each other the total force exerted on each platelet will be f_0 (since $f_0/2$ will come from the reaction force of the first platelet pulling on the second and another $f_0/2$ would be exerted by the other platelet). If the distance between two platelets is such that $f_r \cdot 2r_p < d_{pp} < 2r_p$, the two platelets will be in each other's adhesive zones. Note that in our simulations 45 platelets are initially uniformly distributed in space at an average distance of 25 microns from each other.

Supplementary Note 1.3 - Variations in the number of filopodia per platelet. The number of filopodia for each platelet in specific clot contraction simulations is extracted from a log-normal distribution based on the experimentally observed numbers of filopodia per platelet ($\mu = 1.85, \sigma = 0.27$, see **Figure 5** and the **Results** section in the main text) and two more distributions with a lower number of filopodia (log-normal with $\mu = 0.9$ and $\sigma = 0.19$) and a higher number of filopodia (log-normal distribution with $\mu = 3.7$ and $\sigma = 0.38$. The distribution of platelet filopodia count with larger μ corresponds to a higher degree of platelet activation, while the distribution with smaller values of μ corresponds to a lower degree of platelet activation. When initializing the number of filopodia for a specific platelet in our simulation a random number is generated using one of the distributions mentioned above (e.g. normal, low, or high number of filopodia) and if the number is between 2 and 13 (minimum and maximum number of filopodia per platelet) the integer part of the number is used to determine the number of filopodia for that platelet in our simulation.

Supplementary Note 1.4 - Parallelization and model computational cost. The GPU computational cost remains constant with the increase of the number of fibrin fiber nodes. This is because each node is assigned with a GPU thread to update its position. Since the GPU computing uses the shared memory model as opposed to the CPU parallelization in which the distributed memory model is used, the parallel

communication time of the GPU computing in theory is zero. This is a significant advantage over a CPU implementation in which MPI is typically used for message passing (data transfer). The chief computational bottleneck is the GPU memory size. We note that the limitation of the current GPU implementation is the size of the physical memory of the GPU card. If the fiber network data must be split to store on multiple GPU cards, then additional parallel communication time overhead for communication between different GPU cards is introduced. However, the number of nodes in this model simulation study is on the order of thousands or tens of thousands of nodes and can be stored on a single GPU card.

Moreover, the limited number of CPUs endemic in CPU parallelization would mean that, rather than assigning a single node to each process thread for the relatively simple calculations of forward Euler forces (as is the case in GPU parallelization), optimization of CPU-parallelized code would involve assigning nodes to groups and processing each group of nodes as a CPU thread. The assignment of these groups would not necessarily be straightforward, as it would be platform-specific in its implementation based on the number of available cores and decrease code readability without a clear computational advantage since, in effect, a more granular (node-level) parallelization is natural to this model.

Supplementary Note 2 - Initiation of structure of a fibrin network with platelets

The following sections comprise descriptions of the algorithm for constructing the initial configuration of fiber and platelet nodes and their connections comprising the structure of the model clot. (See Figure 3 in the paper describing different node types in the model.) In brief, **Supplementary Note 2.1** describes the first stage wherein fibrin nodes and platelets are distributed through a spherical domain. Platelets are placed stochastically in a manner that ensures that the spatial density of platelets in the model and experimental clots are the same. Fibrin nodes (representing branch points of fibrin network) are placed stochastically through the domain to maintain the fraction of fibrin nodes that are near platelets and ensure the average distance between the nodes to be close to the average fiber segment length observed in experiments. **Supplementary Note 2.2** describes how the fibrin nodes are assigned fibrin fibers connecting them to each other such that their degree of connectivity (i.e., number of connected neighboring nodes) is the same as the connectivity degree distribution observed in experiments. **Supplementary Note 2.3** describes a process by which the initial conditions from **Supplementary Note 2.2** are rearranged to better match the experimental distribution.

Supplementary Note 2.1 - Platelet and fibrin nodes placement. First, a spherical domain of radius, R, is set. Next, a fiber density, ρ_f, and platelet density, ρ_p, are chosen. These parameters are used to calculate the total number of fibers and platelets. The platelet effective radius, R_P, is chosen and a fixed fiber density parameter is chosen which represents the percentage of the total number of fibers from the distribution $0 < E < 1$ that will be created. In the algorithm, we set $E = 0.975$, resulting in less than 2.5% error in the node degree and fiber density.

A fiber length distribution, X, is chosen based on experimental data, and used to calculate the total number of fibers and their corresponding lengths. A collection of samples, $\{f_s\}$, is chosen from X representing preferred fiber lengths. Samples are drawn until $\sum_s \quad f_s \cdot A_f \geq E \cdot \rho_f$, where A_f is the cross-sectional area of a fiber. A fiber degree distribution, Y, is chosen and used to calculate the number of fiber nodes, N. A parameter, P_{fnp}, is chosen to divide fiber nodes among platelets and the domain. This parameter represents the percentage of fibers near platelets. N is then divided into two parts: the number of fiber nodes near platelets, $N_P = P_{fnp} \cdot N$, and the number of fiber nodes in the domain, $N_F = N(1 - P_{fnp})$. A $P_{fnp} = 0.5$ together with a single filopodia initial force provided us with the best qualitative fitting to the available experimental data.

Platelets are uniformly distributed inside the domain using Latin Hypercube Sampling (LHS) [6,7]. If a platelet, p_j, is placed outside the spherical volume in the LHS sampling procedure or if the platelet is far from all fiber nodes, new uniformly distributed coordinates are chosen. The new coordinates are re-generated until $min_i\ (dist(p_j, f_i)) < R_P$, and $min_{i,i\neq j}\ (dist(p_j, p_i)) > R_P$,where the minimum is taken over all fiber nodes, f_i, and platelets, p_i, respectively. These conditions ensure that all platelets are near some fiber point and far from all other platelet points. Once platelets are placed in the domain, the N_P fiber points are placed so that each point is randomly placed near a platelet within half the distance of the platelet radius, R_P, and the N_F fiber points are randomly placed throughout the spherical domain.

Supplementary Note 2.2 - Degree distribution error reduction. Next, fiber nodes are given a preferred degree, d_i^p, by sampling the distribution, Y. When fibers are generated between nodes, a node is considered to be in need of a neighbor if its current degree, d_i^c, is such that $d_i^p > d_i^c$. Fibers are created until $E \sum_i \quad d_i^p < \sum_i \quad d_i^c$ according to the following algorithm. A node, n_i, with $d_i^p > d_i^c$ is chosen at random to start a fiber. An end node must then be chosen to complete the fiber. All points, n_j, with $d_i = dist(n_i, n_j) < R$ are weighted with weight $X(d_i)$. Next, a single sample s is drawn from X. The node n_j satisfying $min|X(d_i)\ -\ s|$ is chosen to end the fiber if the fiber between n_i and n_j has not yet been established. If the fiber has already been established, we set $X(d_i) = 0$ and choose a new sample s^* from X, resulting in a new end point for the fiber beginning at n_i. If no node can be found, a new random starting node n_i is chosen again from the nodes such that $d_i^p > d_i^c$. This process of creating fibers continues until the desired density of fibers is reached. Choosing fibers in this manner serves to approximate the target distributions for experimental fiber length and node degree.

Supplementary Note 2.3 - Length distribution error reduction. To increase the accuracy between the current fiber lengths and$\{f_s\}$, nodes are perturbed randomly within the domain. A random move serves to slightly change the associated fiber lengths. If a point from N_F is chosen, any move is allowed within the fixed domain. If a point, n_i, from N_P is chosen, the random perturbation must satisfy $dist(n_i, P) < R_P$, where P is the platelet associated to n_i. To determine fiber length error, a sample is drawn representing each fiber from the experimental distribution. Samples are binned with width (=0.1) and errors are determined by comparing the frequency in each bin. Using this method, a node perturbation is accepted if the new fiber lengths lower the fiber length error.

Supplementary Note 3 - Model calibration

Supplementary Note 3.1 - Calibration of an individual fiber sub-model. Fibrin fiber elastic properties were calibrated using the general WLC modeling approach. The WLC model has been previously shown to provide analytical and theoretical explanations for single fibrin fiber mechanical behavior [8]. Here, we use the experimental stress-strain curves for a single fibrin fiber obtained using atomic force microscopy (AFM) [5] to calibrate each simulated fiber response to stretching. Two parameters, persistence length and contour length multiplier, in the WLC model were determined by using a recursive least-squares algorithm [9]. The experimental data and resulting fitting curve for a single fiber are shown in **Supplementary Figure 1**.

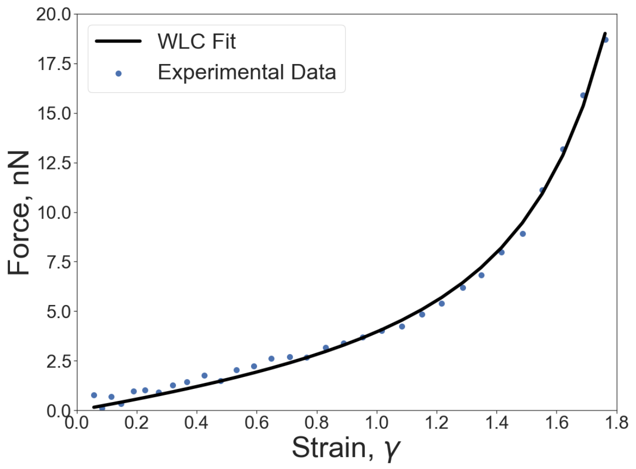

Supplementary Figure 1. Single fiber force-strain response calibration. Black line shows the model fit to experimental data for single fiber (blue dots) force-strain response using an atomic force microscope AFM [5].

Supplementary Table 2 lists parameter values that are calculated based on the recursive least-squares optimization under the assumption that the temperature is fixed at 300 Kelvin and 200-1100 monomers would fit in the circular cross-sectional area of a fiber [5].

**Supplementary Table 2**: Fibrin Fiber Model Simulation Parameters	
Monomers Per Fiber, N_m	200-1100 [5]
Monomer Persistence Length, L_p	0.005-1.074 nm [5]
Absolute Temperature, T	300 K
Normalized Contour Length Multiplier, C	2.350 [5]

To incorporate fiber bending, bending springs are added between sub-nodes for all fibers (see **Figure 3B** in the main text). A bending spring is placed at every node triplet (see **Figure 3B** in the main text). Deviation from an equilibrium angle results in generation of a restoring force that is calculated with the bending spring potential (Eqn. 11). The bending spring constant, B_s, is calculated using the relation $B_s = EI$, where E is the Young's modulus based on the linear response of individual fibers, and I is the moment of inertia for a circular shape. Assuming all fibers maintain the same diameter of 100 nm, we can calculate B_s = 2.252×10^{-3} nNμm^2.

Supplementary Note 3.2 - Calibration of individual platelet model. We implemented the following representation of the platelet contractile force:

$$F_{platelet}\,(t,k) = f_0 + (1 - de^{-t/\tau})\frac{ak}{bk+c} \qquad (12)$$

where $k = dF_{WLC}/d\gamma = NK_bT/P(C^2/(2(C-\gamma)^3 + 1/C)$, f_0 is the force developed at the absence of substrate (or when k=0), γ - fiber strain, F_{WLC} is the WLC (worm-like chain fiber force response model,

τ - force relaxation time. The term $(ak/(bk + c))$ was introduced in [10] while $(1 - exp\,(-t/\tau))$ is a loading time factor and a,b,c are fitting coefficients. Formula (12) takes into account both exponential loading time observed in [11] and the monotonic increase and saturation of the contractile cellular force, similarly to what was observed in [10] where stem cells forces have shown to have a comparable dependence on stiffness of their surrounding extracellular matrix.

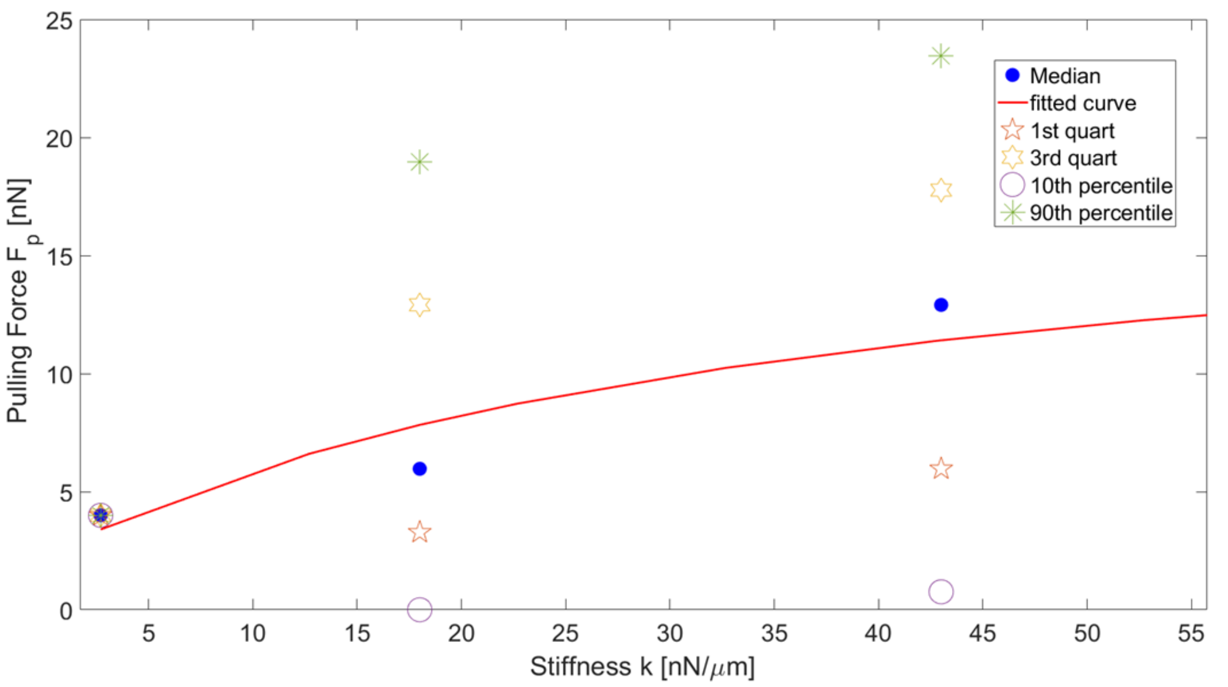

Supplementary Figure 2. Force fitting at t=∞, when the cantilever reaches equilibrium (see also [11]).

Based on data from Lam et al.[11], the parameters in the equation (12) are obtained through least square fitting as follows. First, we used the data in Lam's paper Figure 2C and a minimum force of $4nN$at k_0 (no strain) to fit a,b,c,f_0 using least square fitting with $F_{platelet}\,(t,k) = f_0 + \frac{ak}{bk+c}$ that is obtained from formula (12) for platelet force at t=∞, when the cantilever reaches equilibrium (see also [11]). This resulted in the values $a = 61$, $b = 3.6$, $c = 130$, *and* $f_0 = 2.2$ (see also **Supplementary Figure 2** and **Supplementary Table 3**). Second, we use the data in supplementary material of Lam's paper figure 1A with $k = 18\,nN/\mu m$ (chosen because the typical range of stiffness in our simulations is $[2.71, 20.81]nN/\mu m)$ and the values of a,b,c,f_0 previously determined to find d and τ obtained using least square fitting for formula (12). This resulted in the values $d = 1.4$ and $\tau = 640$ (see also **Supplementary Figure 2**). However, equation (12) cannot be used as is since Lam's data refers to a platelet attached on two sides to a cantilever and a stiff surface. To adapt it for use with filopodia we proposed the following modifications

$$F_{filopod}\,(t,k) = (f_0 + \frac{ak}{bk+c})/2 \qquad (13)$$

$$F_{filopod}\,(t,k) = (f_0 + (1 - de^{-t/\tau})\frac{ak}{bk+c})/2 \qquad (14)$$

$$F_{filopod}\,(t,k) = (f_0 + \frac{ak}{bk+c}) \qquad (15)$$

$$F_{filopod}\,(t,k) = (f_0 + \frac{ak}{bk+c})/N_p(t) \qquad (16)$$

with $N_p(t)$ representing the number of filopodia pulling at time t. Equations (13) and (14) both assume that the measured data of platelet force from Lam et al.[11] comprise forces on two sides of the platelet, and that only a single filopod will act on each side (hence the division by 2). However, equation (13) also assumes instantaneous force loading time for the filopodia. Similarly, equation (15) also assumes instantaneous loading time but does not divide the force by 2. Finally, equation (16) assumes that the total force of the platelet of equation (12) is instantaneously loaded and equally divided between active platelets at time t.

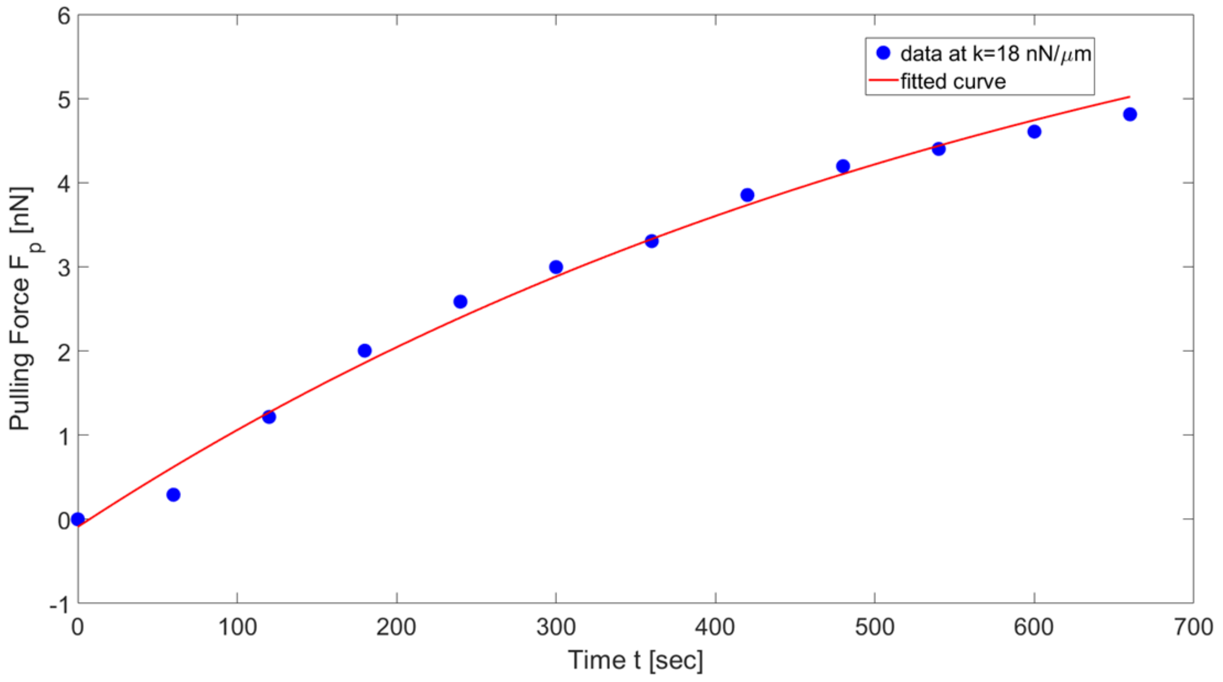

Supplementary Figure 3. Force fitting at fixed stiffness in our stiffness range, [2.71,20.81] nN/μm.

Formula (13) provided us with the best agreement with experimental data (see **Supplementary Figure 3**; further demonstrated in **Supplementary Figure 6**). The transition from the full-platelet force expression (12) to the single-filopod expression (13) is also mechanistically reasonable. In the experimental settings of [11], the force on a platelet has been measured between the two broad surfaces of a cantilever and the substrate, while the filopodia can have only one point of adhesion (i.e., one filopod to one fiber). Therefore, the force measured in these experiments may be the result of the platelet exerting force with two filopodia - one attached to the surface and another to the cantilever. This suggests that the force for one filopod should be expressed as half of what is given in formula (12), if indeed (12) was the magnitude of force measured from two filopodia.

Furthermore, during active contraction, platelet filopodia contract much faster than the platelets in the atomic force microscopy experiments [11]. Therefore, the model assumes a fast loading time by setting the time t equal to infinity in equation (12) and dividing the force by two because the filopodia has only one focal point for the exerted force instead of two like in [11].

Supplementary Table 3: Platelet Contractile Force Parameter Values [11]

f_0, force of platelet for zero stiffness	2.2 nN
a, fitting parameter	61
b, fitting parameter	3.6
c, fitting parameter	132
d, fitting parameter	1.4 nN
τ, force relaxation time	637 seconds

Supplementary Note 4 - Supplementary results

Supplementary Note 4.1 - Model validation. The change in fiber density over time from **Figure 7C** is fitted with a polynomial function and then the most relevant peaks (non-negligible maxima) of the fitted polynomials are selected as the beginning and ending time of the kinetic clot contraction phases. The exact numbers are reported in **Table 1** of the main text together with the average contraction rates in the phases calculated as the mean change in fiber density during each phase.

Supplementary Note 4.2 - Impact of the number of platelet filopodia on the clot contraction kinetics. The change in fiber density over time from **Figure 8C** is fitted with a polynomial function and then the most relevant peaks (non-negligible maxima) of the fitted polynomials are selected as the beginning and ending time of the kinetic clot contraction phases. The exact numbers are reported in **Table 1** of the main text together with the average contraction rates in the phases calculated as the mean change in fiber density during each phase.

Supplementary Note 4.3 - Clot contraction kinetics. Platelets have been observed to sense and dynamically respond to variations in the clot microenvironment, such as alterations in substrate stiffness and locally applied forces [11-13]. While individual cellular reactions may vary, the general consensus reveals a correlation between increased substrate stiffness and increased expression of the active integrin $\alpha IIb\beta 3$, platelet spreading, and contractile force [12]. In this section, we present several different simulated responses of platelets due to changes in the stiffness of the extracellular matrix and compare the contraction metrics obtained, with a particular focus on the densification rate (see **Supplementary Figure 6C**) and the contraction phases observed in experiments. In particular, we tested variations of the filopod force obtained in **Supplemental Note 3.2**. We compared simulations with filopodia forces given by equation (13) and the other force types obtained from the general equation (12) (see **Supplemental Note 3.2**). These forces were obtained for different hypothesized force loading rates (i.e., how long it takes a platelet from the beginning of contraction to achieve full pulling force) or concerning the division of the platelet force among active filopodia (i.e., are the filopodia independent or dependent on each other).

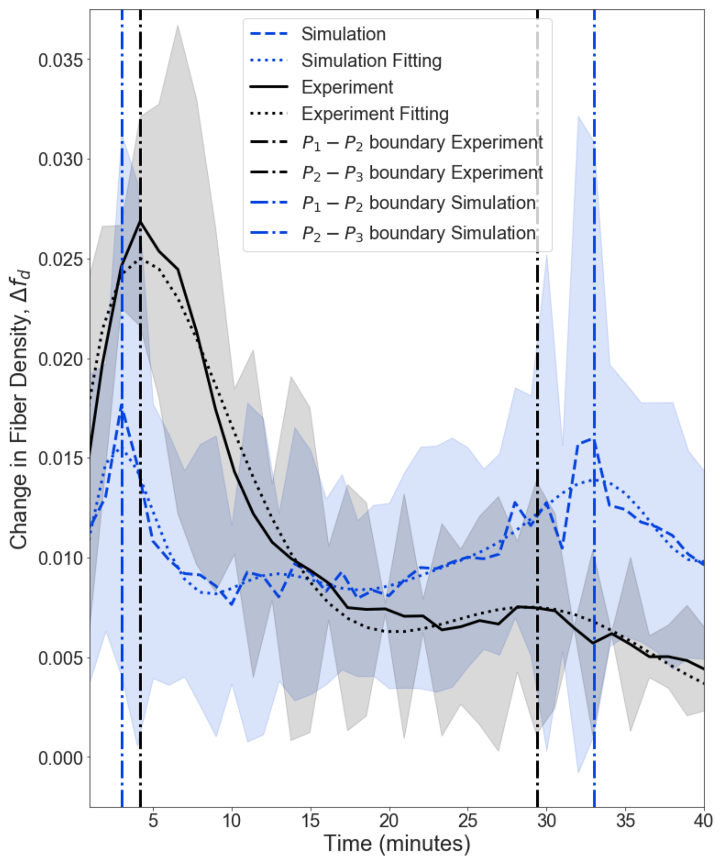

Supplementary Figure 4. Calculation of boundary of kinetic phases of clot contraction. The change in fiber density over time from **Figure 7C** is fitted with a polynomial function and then the most relevant peaks (non-negligible maxima) of the fitted polynomials are selected as the boundary of the kinetic clot contraction phases. The black lines indicate Experimental results, and the blue lines indicate Simulation results. The original data from both simulations and experiments is indicated as in **Figure 7C.** The dotted lines are the polynomial fittings. The dashed-dotted lines indicate the boundaries between phases. The shaded region around each line shows the standard deviation corresponding to each value.

For each type of response, 10 simulations were performed. Simulations using equations (13) and (15) (**Supplementary Figure 6** blue dashed line and green dotted line) showed a closer agreement with experimental results (**Supplementary Figure 6**). Moreover, for these simulations, the rate of fiber densification revealed several phases that qualitatively agree with the densification phases observed in experiments [14]. In particular, in these two simulations, it is possible to observe a growth in the densification rate (phase P_1), a decrease (phase P_2), and an asymptotic limit (phase P_3).

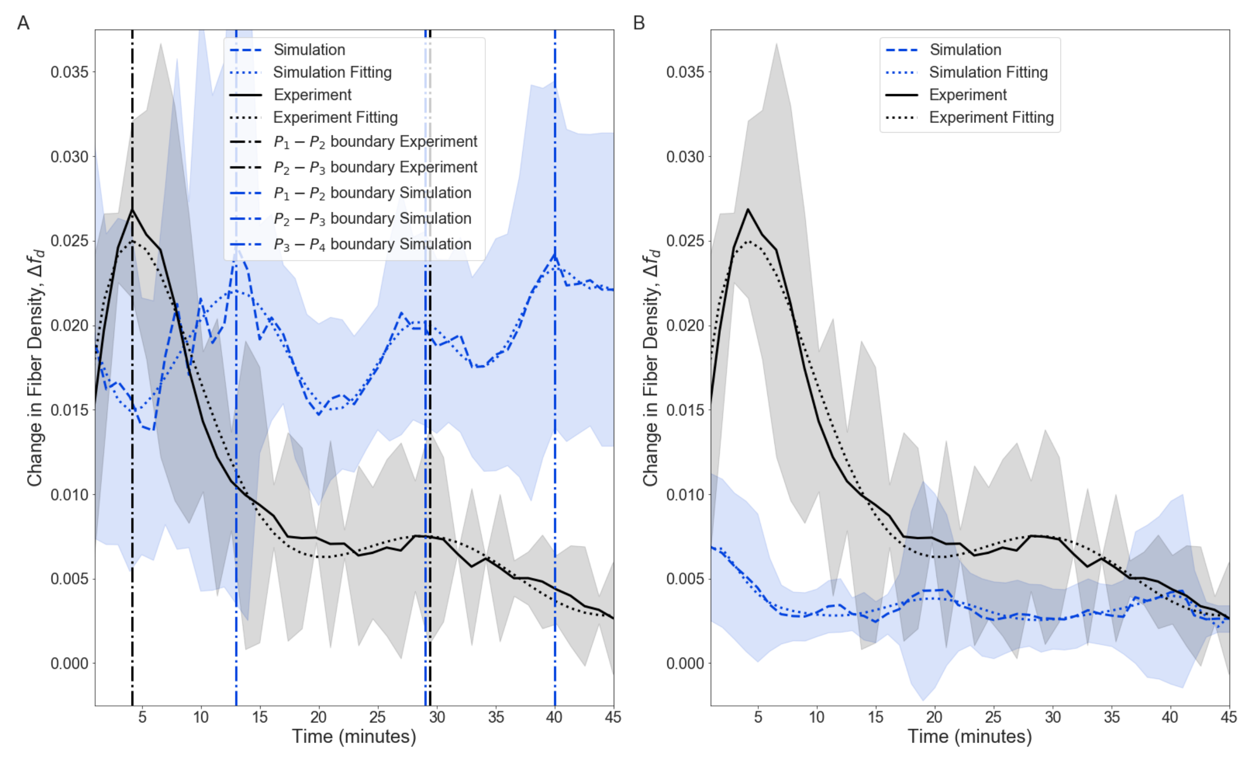

Supplementary Figure 5. Calculation of boundary of kinetic phases of clot contraction for simulated clots with increased number of filopodia (A) and decreased number of filopodia (B). The change in fiber density over time from **Figure 8C** is fitted with a polynomial function and then the most relevant peaks (non-negligible maxima) of the fitted polynomials are selected as the boundary of the kinetic clot contraction phases. The black lines indicate Experimental results, and the blue lines indicate Simulation results. The original data from both simulations and experiments is indicated as in **Figure 8C.** The dotted lines are the polynomial fittings. The dashed-dotted lines indicate the boundaries between phases. The shaded region around each line shows the standard deviation corresponding to each value.

These simulations revealed that even similar force profiles (based on equation (12)) can produce significantly different results. Also, we observed that the force described by equation (13) is the one that best reproduces the number and timing of contraction phases observed in experiments (see **Supplementary Figure 6**).

Supplementary Note 4.4 - Platelet clustering during clot contraction. Supplementary Figure 7 shows projections on the x-y plane of the positions of single platelets and centers of mass of clusters containing from 2 to 6 platelets at 0, 10, and 30 minutes. The figure shows how clusters containing 2-6 cells form in a clot volume at different times during clot contraction. Model simulations show that the platelet clusters (i.e., groups of two or more touching platelets) are distributed uniformly throughout the network with respect to the angle from the center of the clot (see **Supplementary Figure 7** and **Supplementary Figure 9**).

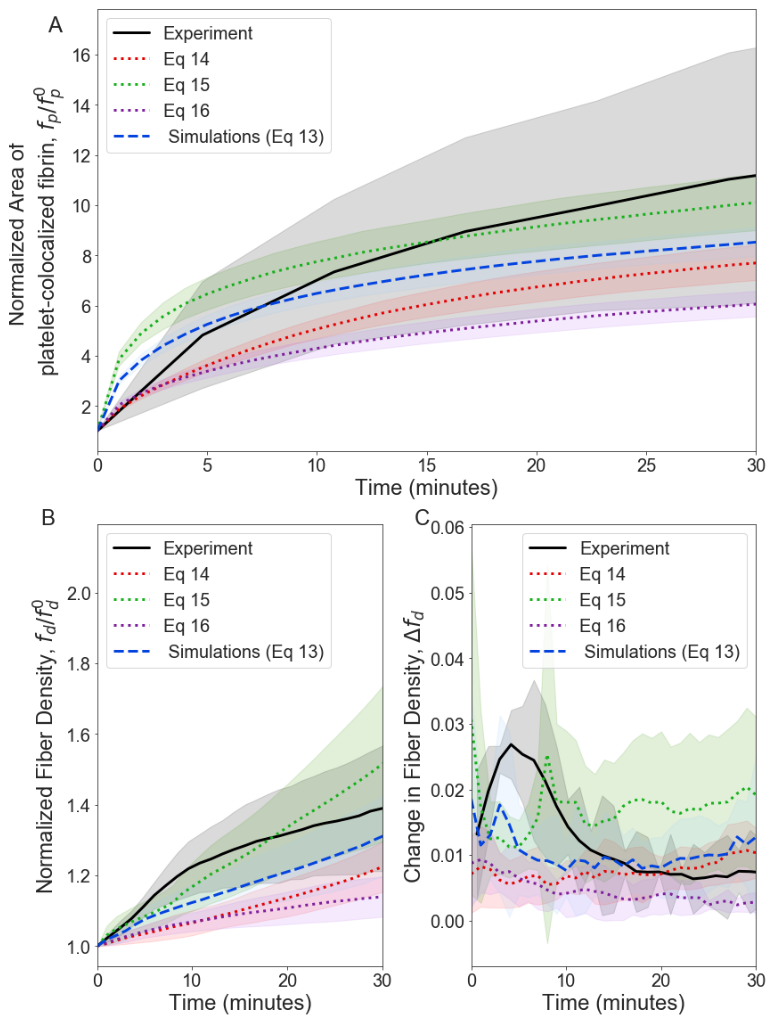

Supplementary Figure 6. Fibrin fiber compaction and densification during clot contraction at various filopodia responses to fiber stiffness. Standard simulations from equation (13) (*dashed blue line*) and experimental data (*solid black line*) are contrasted with simulations obtained using equations (14), (15), and (16) for the filopodia pulling force (*dotted lines, red, green and purple respectively*). (A) Normalized area of platelet-colocalized fibrin, $f_{platelet}$. (B) Fibrin density, f_{dense}, in the central 10 μm of the fibrin network as a function of time. (C) Change in fibrin density, $\Delta f_{dense}, over\ time$. Simulations were performed for 30 minutes of real-time (M ± STD, n=10). The shaded region around each line shows the standard deviation corresponding to each value.

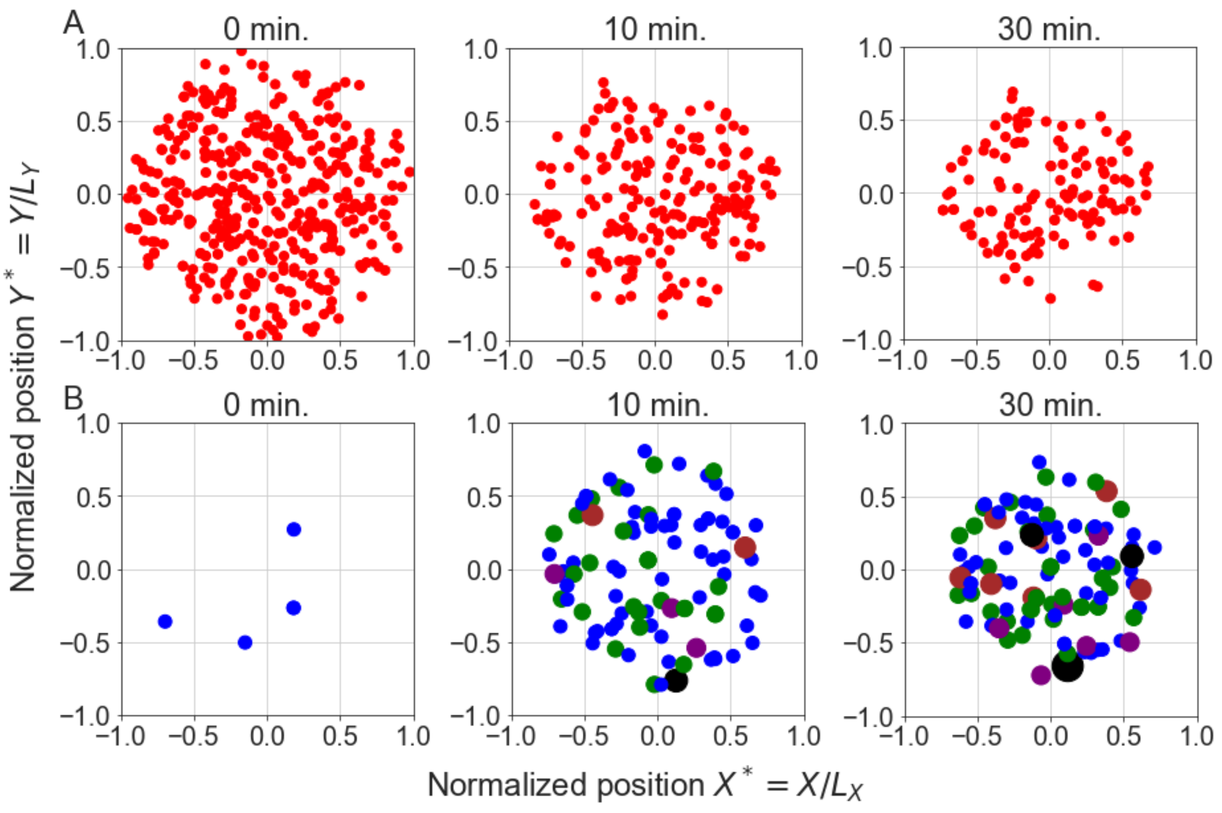

Supplementary Figure 7. Temporal changes in the spatial distribution of platelet clusters and single platelets within a contracting clot. Representative 2D projections (x-y plane) of single platelet (A) and platelet cluster (B) coordinates at different time points during clot contraction. Single platelets are shown in red. Platelet clusters containing 2-6 platelets are shown in different colors: two-platelet clusters are in blue, three in green, four in purple, five in brown, and six in black. The results are based on combined 10 simulations with 45 platelets per clot in each simulation.

The speed of platelets during clot contraction was evaluated. These simulation results allow us to determine if the platelet positions are tending toward equilibrium (i.e., speed decreases) as the contraction progresses (i.e., higher simulation time). This relates to the formation of clusters, in that as platelets slow down, it takes longer for them to approach each other and for clusters, and therefore platelets clusters form less at later simulation times. As construction progresses and elastic forces in the fibrin network reach an equilibrium with the platelets pulling forces, the platelets start moving less and the clot approaches equilibrium, as can also be deduced by the slowdown in contraction rates observed in **Supplementary Figure 4**. Note that as our system is in an overdamped regime a low velocity corresponds to approaching an equilibrium of forces. **Supplementary Figure 8** shows the probability density of the speed of platelet relocation during clot contraction at 10, 20, 30, and 40 minutes. As the clot contracts, the fraction of slower moving platelets increases and, as a consequence, platelets form clusters more slowly as clot contraction progresses.

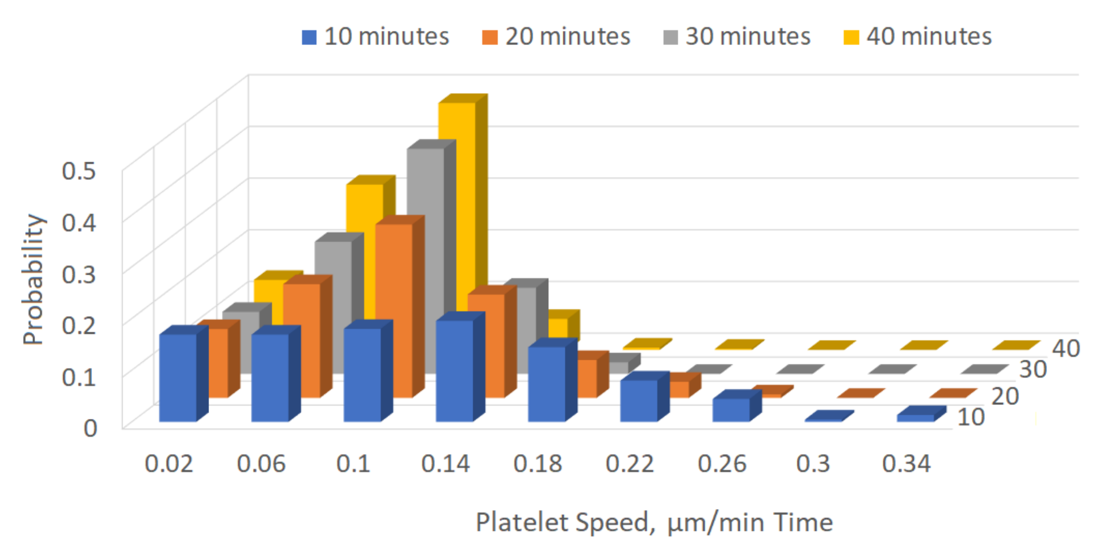

Supplementary Figure 8. Probability distribution of speeds of platelet relocation in contracting clots. Normalized histograms of the number of platelets moving at various speeds at different time points of clot contraction. Time points: 10 (*blue*), 20 (*orange*), 30 (*grey*), 40 (*yellow*), and 40 minutes. Speeds range from 0.02 to 0.4 μm/min.

Supplementary Figure 9 shows the angle distribution in the x-y plane of the platelet clusters in our simulated clot at t=10, 20, 30, and 40 minutes of clot contraction. This angle is calculated with respect to the x-axis in our simulation coordinate axis and its relative angle with respect to the projection on the x-y plane of the line connecting the center of our clot and the center of a cluster of platelets. We can observe that two-platelet clusters are equally distributed with respect to their angle in the spherical clot. Moreover, bigger size clusters, while less in number, do not show any marked asymmetric distribution (see also **Supplementary Figure 7**). Other 2D projections (x-z and y-z plane) show similar pictures. **Supplementary Figure 9** demonstrates that no radial asymmetry forms in the spatial distribution of platelets clusters in our model.

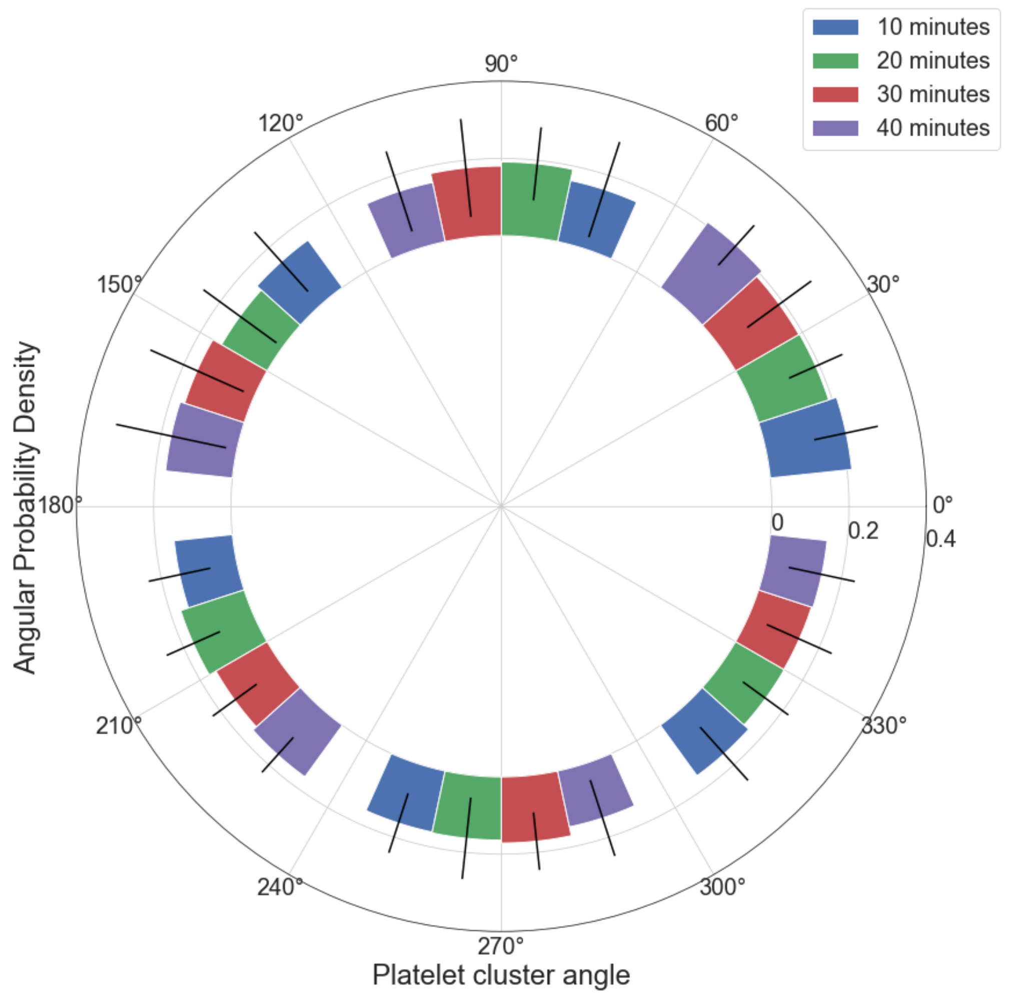

Supplementary Figure 9. Angular distribution of platelet clusters at various times during clot contraction. The angular probability density function of platelet clusters with 2 or more platelets in the x-y plane is shown by a pie chart. Different colors correspond to different time moments of clot contraction in simulations. The black bars represent the error as one standard deviation from the mean indicated with the corresponding-colored histogram bar.

Supplementary Note 4.5 - Additional analysis of fibrin strain. Supplementary Figure 10 shows the evolution of strain in time across multiple simulated clots. Looking at the mean strain per fibrin segment will show only the mean and standard deviation of up to strain 0.1 at approximately 40 minutes. It may be important to note that in **Figure 7**, we observe fiber strain of up to 1.8 localized into bundles between platelet clusters. Additional simulations were run wherein we reduced the force magnitude that filopodia exert on fibrin to one-tenth of experimentally calibrated values to replicate the effects of a non-muscle myosin IIA inhibitor treatment. Our quantifications of this effect are shown in **Supplementary Figure 11**.

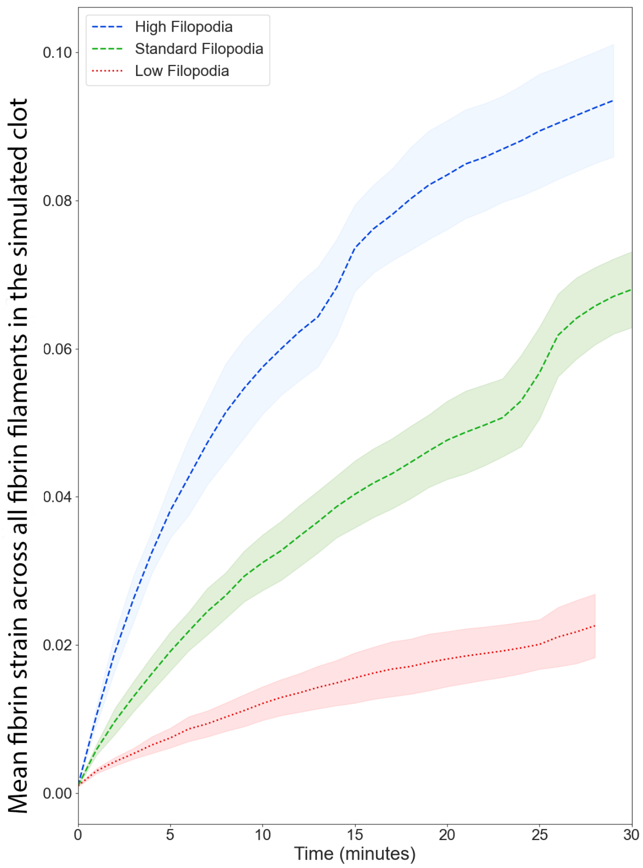

Supplementary Figure 10. The means and standard deviations of mean-strain-per-node for different types of simulated clots are shown. Each dotted line and ribbon shows the average value and standard deviation of mean-strain-per-filopodia across ten simulated clots. Simulations with experimentally observed intermediate filopodia numbers are shown by the green line, while blue dashed lines and red dotted lines represent average strain with the higher and lower filopodia numbers, respectively.

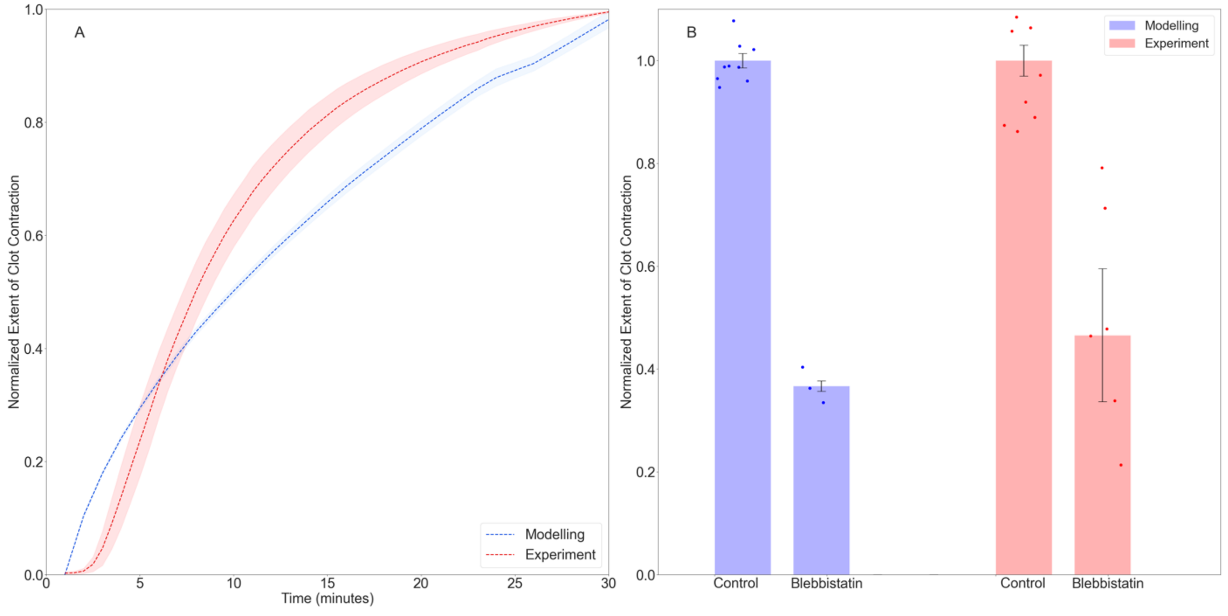

Supplementary Figure 11. (A) Contraction kinetic curves for simulated and experimental *in vitro* PRP clots showing temporal changes in the extent of clot contraction normalized by its final value. (B) Relative changes in the extent of clot contraction for simulated (Control: n=10, Blebbistatin n=3) and *in vitro* (Control n=8, Blebbistatin n=6) PRP clots in the presence of myosin IIA inhibitor, blebbistatin, at 30 min of incubation (M±SEM).

Supplementary References

1 Kubo, R. The fluctuation-dissipation theorem. *Reports on Progress in Physics* **29**, 255-284 (1966). https://doi.org:10.1088/0034-4885/29/1/306

2 Britton, S. *et al.* Contribution of nascent cohesive fiber-fiber interactions to the non-linear elasticity of fibrin networks under tensile load. *Acta Biomater* **94**, 514-523 (2019). https://doi.org:10.1016/j.actbio.2019.05.068

3 Chwang, A. T. & Wu, T. Y.-T. Hydromechanics of low-Reynolds-number flow. Part 2. Singularity method for Stokes flows. *Journal of Fluid Mechanics* **67**, 787-815 (1975). https://doi.org:10.1017/s0022112075000614

4 Miller, C. C. The Stokes-Einstein law for diffusion in solution. *Proceedings of the Royal Society of London. Series A, Containing Papers of a Mathematical and Physical Character* **106**, 724-749 (1924). https://doi.org:10.1098/rspa.1924.0100

5 Houser, J. R. *et al.* Evidence that αC Region Is Origin of Low Modulus, High Extensibility, and Strain Stiffening in Fibrin Fibers. *Biophysical Journal* **99**, 3038-3047 (2010). https://doi.org:10.1016/j.bpj.2010.08.060

6 McKay, M. D., Beckman, R. J. & Conover, W. J. Comparison of Three Methods for Selecting Values of Input Variables in the Analysis of Output from a Computer Code. *Technometrics* **21**, 239-245 (1979). https://doi.org:10.1080/00401706.1979.10489755

7 Marino, S., Hogue, I. B., Ray, C. J. & Kirschner, D. E. A methodology for performing global uncertainty and sensitivity analysis in systems biology. *Journal of Theoretical Biology* **254**, 178-196 (2008). https://doi.org:10.1016/j.jtbi.2008.04.011

8 Hudson, N. E. *et al.* Stiffening of Individual Fibrin Fibers Equitably Distributes Strain and Strengthens Networks. *Biophysical Journal* **98**, 1632-1640 (2010). https://doi.org:10.1016/j.bpj.2009.12.4312

9 Young, P. 42-54 (Springer Berlin Heidelberg., 1984).

10 Zemel, A., Rehfeldt, F., Brown, A. E. X., Discher, D. E. & Safran, S. A. Optimal matrix rigidity for stress-fibre polarization in stem cells. *Nature Physics* **6**, 468-473 (2010). https://doi.org:10.1038/nphys1613

11 Lam, W. A. *et al.* Mechanics and contraction dynamics of single platelets and implications for clot stiffening. *Nature Materials* **10**, 61-66 (2011). https://doi.org:10.1038/nmat2903

12 Qiu, Y. *et al.* Platelet mechanosensing of substrate stiffness during clot formation mediates adhesion, spreading, and activation. *Proceedings of the National Academy of Sciences* **111**, 14430-14435 (2014). https://doi.org:10.1073/pnas.1322917111

13 Myers, D. R. *et al.* Single-platelet nanomechanics measured by high-throughput cytometry. *Nature Materials* **16**, 230-235 (2017). https://doi.org:10.1038/nmat4772

14 Kim, O. V., Litvinov, R. I., Alber, M. S. & Weisel, J. W. Quantitative structural mechanobiology of platelet-driven blood clot contraction. *Nature Communications* **8** (2017). https://doi.org:10.1038/s41467-017-00885-x
